# Supplementary material for: Dipole field in nitrogen-enriched carbon nitride with external forces to boost the artificial photosynthesis of hydrogen peroxide
Source: Nat Commun. 2023 Sep 16;14:5742. doi: 10.1038/s41467-023-41522-0 (PMC10505161; doi:10.1038/s41467-023-41522-0)
Supplement: Supplementary file 1 — Supplementary information [file 41467_2023_41522_MOESM1_ESM.pdf]

Supplementary Information

(12 Texts, 61 Figures, 5 Tables and 64 References)

Dipole field in nitrogen-enriched carbon nitride with external forces to boost the artificial photosynthesis of hydrogen peroxide

Zhi Li[1], Yuanyi Zhou[1], Yingtang Zhou[2], Kai Wang[3], Yang Yun[4], Shanyong Chen[1], Wentao Jiao[5,*], Li Chen[6], Bo Zou[3], Mingshan Zhu[1,*]

[1] Guangdong Key Laboratory of Environmental Pollution and Health, School of Environment, Jinan University, Guangzhou 511443, PR China

[2] Marine Science and Technology College, Zhejiang Ocean University, Zhoushan 316004, China.

[3]State Key Laboratory of Superhard Materials, College of Physics, Jilin University, Changchun 130012, China.

[4]College of Environment and Resource, Research Center of Environment and Health, Shanxi University, Taiyuan 030006, China.

[5]Research Center for Eco-Environmental Sciences, Chinese Academy Sciences, Beijing 100085, China.

[6]Department of General Practice, First Medical Center, Chinese PLA General Hospital, Beijing, 100853, China.

Correspondence and requests for materials should be addressed to W.J. (wtjiao@rcees.ac.cn) or M.Z. (zhumingshan@jnu.edu.cn).

Supplementary Texts

Text S1. Materials.

3-Amino-1,2,4-triazole (3-AT, $C_2H_4N_4$, ≥ 96.0%), 5,5-dimethyl-1-pyrroline (DMPO), p-benzoquinone (p-BQ) and potassium hydrogen phthalate ($KHC_8H_4O_4$) were purchased from Aladdin Chemical Reagent Co., Ltd., China. Melamine ($C_3N_3(NH_2)_3$, ≥ 99.0%), tert-butanol (TBA), Potassium iodide (KI), ammonium molybdate $(NH_4)_6Mo_7O_{24}\cdot 4H_2O)$, Sulfuric acid ($H_2SO_4$), Sodium hydroxide (NaOH), Sodium sulfate (Na_2SO_4) and ethanol (EtOH) were purchased from Sinopharm Chemical Reagent Co., Ltd. All the reagents were of analytical grade and were used without further purification.

Text S2. Instruments.

The morphology was measured by Transmission Electron Microscope (TEM, JEOL 2010F). The crystal structure of powder product was investigated via X-ray diffraction (XRD) D2 PHASER with Cu-Kα radiation with 2-theta degree from 10 to 70. Fourier transform infrared spectrophotometer (FT-IR) (IRTracer-100, Shimadzu, Japan) was used to explore organic structure. The optical absorption behaviors were measured by UV-Vis spectrophotometer (JASCO V-770, Japan). Thermo Scientific K-Alpha X-ray photoelectron spectroscopy system (Thermo Fisher Scientific, UK) was used to detect XPS signals. Piezoresponse force microscopy (PFM) with a scanning probe mode (Asylum Research, Nanoworld) was used to characterized the piezoelectric response of samples. The photo-assisted KPFM measurement was also conducted in the AFM test system equipped with SKPM module and Xenon lamp. The O_2 adsorption on the samples were performed by temperature-programmed desorption (TPD, DAS-7000). The as-prepared samples (50 mg) was heated to 150 °C under pure N_2 with a heating rate of 10 ^oC min^{-1}, and maintained at 150 ^oC for 1 h. After cooling the sample to room temperature, adsorption of O_2 was carried out in flowing O_2 (30 mL min^{-1}) for 1 hour. And the O_2-TPD measurement was tested in flowing pure N_2 with a heating rate of 10 ^oC min^{-1} until 500 °C. Desorbed O_2 was monitored by thermal conductivity detector. The Raman spectra were recorded at room temperature with LabRAM HR Evolution

spectrometer. The chemical structure of sample was further proved using organic elemental analysis (UNICUBE-Elementar), Matrix-assisted laser desorption/ionization–time of flight mass spectrometry (MALDI-TOF-MS, Bruker Autoflex Speed TOF/TOF), liquid chromatography time-of-flight mass spectrometer (LC-TOF-MS, AB Sciex Triple TOF 5600) and near-edge X-ray absorption finestructure (NEXAFS) at the beamline BL14W1 station of the Beijing Synchrotron Radiation Facility, China.

Text S3. Organic elemental analysis.

We evaluate the elemental ratio of C to N for all samples using organic elemental analysis. The CHN results of C_3N_5 (CN-500°C) confirm that the average weight percentages of C and N are 32.6 % and 62.6 %, respectively (**Table S3**). The average atomic rate of C/N is thus determined to be 0.607, which is very close to the theorical value (0.60), confirming the successful synthesis of C_3N_5. Specifically, for 3-AT and CN-200°C, the elemental proportion of CNH has barely changed, which may be due to the fact that the boiling point of 3-AT is 244.9°C, implying that the chemical composition has not changed. For CN-300°C, CN-400°C and C_3N_5 (CN-500°C), with the increase of synthesis temperature, the percentage weight of N and H decreases gradually, this may be due to the possible release of ammonia and hydrogen during the synthesis process. The total mass fraction of 3-AT exceeded 100%, due to the water absorption of the sample.

Text S4. Nuclear magnetic resonance (NMR) spectra.

The ^{15}N spectra of 3-AT and CN-200°C were tested with liquid NMR analysis (JNM-ECZ400R with 5mm Royal probe) dissolved by DMSO. The ^{13}C solid-state NMR spectra of all samples and ^{15}N solid-state NMR spectra of CN-300°C, CN-400°C, C_3N_5 (CN-500°C) and C_3N_4 were acquired on Bruker ADVANCE III 400 and 600 equipped with a 4 mm double resonance MAS NMR probe using the cross-polarization magic-angle spinning (CPMAS). ^{13}C spectra were referenced to TMS ($\delta(^{13}C)$ = 0.00 ppm) by setting the high frequency ^{13}C peak of solid adamantane to 38.56 ppm. ^{15}N spectra were referenced to nitromethane $\delta(^{15}N)$ = 0.00 ppm by setting the isotropic peak of a glycine sample (98 % ^{15}N) to −347.6 ppm.

Text S5. *E. coli* disinfection.

The bacteria suspension of *E. coli* (BW-2533) was prepared according to previous report.[1] Briefly, 1 mL *E. coli* suspensions containing different initial concentrations (1000 CFU mL^{-1} and 10000 CFU mL^{-1}) were prepared. Bacterial inactivation experiments were subsequently carried out using generated H_2O_2 after filtering the photocatalyst particles. 25 µL filtrate solution containing generated H_2O_2 was taken every 20min for adding to the bacterial suspension, and subsequently grown aerobically in stationary phase at 36°C for 24 h. The inhibition ratio was determined by a plate count method according to the culturable bacterial density.

Text S6. Photocatalytic hydrogen evolution with ultrasonic force

The photocatalytic H_2 evolution tests were performed on an online photocatalytic H_2 evolution system with visible light and ultrasonic force. Before testing, N_2 was flooded throughout the reaction system for 30min. In detail, 5 mg of C_3N_4 and C_3N_5 was suspended in 10 mL of deionized water containing ethanol (10 vol%). At certain time intervals, the resulting gas were analyzed by a gas chromatograph (GC-2014c, SHIMADZU, Japan) with a thermal conductivity detector (TCD) for H_2 detection. High purified N_2 (99.999%) and H_2 (99.999%) were used as carrier gas.

Text S7. AQE measurements

AQE measurements: For AQE measurements, 20 mg of photocatalyst was dispersed in 40 mL of 10 vol% EtOH. A 300 W Xe-lamp with a band-pass filter of 380±15 nm, 420±15 nm, 450±15 nm, 500±15 nm, 550±15 nm, 600±15 nm or 650±15 nm was used as the incident light source. The light intensity was adjusted to be 4.52 mW cm^{-2}, 3.13 mW cm^{-2}, 3.64 mW cm^{-2}, 6.23 mW cm^{-2}, 7.07 mW cm^{-2}, 6.68 mW cm^{-2} and 7.74 mW cm^{-2}, respectively. The irradiation area was controlled to be 4.9 cm^2. The amount of H_2O_2 production was analyzed after 1 h irradiation. AQE was calculated using the following equation:

$$AQE\% = 2 \times (N_{H_2O_2} \cdot N_A \cdot h \cdot c) / (I \cdot S \cdot t \cdot \lambda) \times 100\%$$

where $N_{H_2O_2}$ was the amount of H_2O_2 production (mol), N_A was the Avogadro

constant (6.022×10^{23} mol^{-1}), *h* was the Planck constant (6.626×10^{-34} J·s), *c* was the speed of light (3×10^{8} m·s^{-1}), *I* was the irradiation intensity (W·cm^{-2}), *S* was the irradiation area (cm^{2}), *t* was the irradiation time (s) and *λ* was the wavelength of incident light (m).

Text S8. Electrochemical measurements

The ORR performances were evaluated on a CHI760E electrochemical workstation using a three-electrode configuration electrochemical cell. The Ag/AgCl electrode and graphite rod were used as the reference electrode and counter electrode, respectively. The rotating ring disk electrode (RRDE, PINE Research Instrumentation, electrode area: 0.2475 cm^{2}) was employed as the work electrode. The typical catalyst ink was prepared was prepared as following. Firstly, 4 mg of the catalysts and 30 μL of Nafion (5 wt.%) were added into the 0.97 mL of water/ethanol mixing solution (V_{water}:$V_{ethanol}$=10:87) and then were dispersed by sonication for 1 h to form a homogeneous ink. Lastly, 5 μL of the ink was dropped on the polished RRDE and dried at room temperature (catalyst loading: 0.08 mg cm^{-2}).

Prior to the ORR, CV measurement at the scan rate of 50 mV/s for 40 cycles was conducted on RRDE to electrochemically clean it until stable CV curves could be achieved. The ORR polarization curves were collected by linear sweep voltammograms (LSV) measurement in the O_2 saturated electrolyte with the sweep rate of 10 mV s^{-1} at 1600 rpm. The onset potential is defined as the potential corresponding to the ring current (I_R) of 0.01 mA (5% of the theoretical limiting current).[2, 3] To detect the produced H_2O_2, potential on Pt ring was held at 1.2 V (*vs.* RHE) during LSV. For the chronoamperometry measurement, the potential was fixed at 0.65 V (*vs*. RHE). The collection efficiency (N) was calculated to be 37%. All the LSV curves were corrected by resistance compensation and were referred to the reversible hydrogen electrode (RHE).[4]

The H_2O_2 selectivity and the transfer number electron (*n*) were determined by the disk current (I_D) and ring current (I_R) results according to the following equations:[6]

$$H_2O_2\% = 200\frac{I_R/N}{I_D+I_R/N} \quad (1)$$

$$n = 4\frac{I_D}{I_D+I_R/N} \quad (2)$$

Text S9. Ultrafast transient absorption (TA) spectra

The TA spectra were measured by the pump and probe method using a regeneratively amplified titanium sapphire laser (Spectra-Physics, Spitfire Pro F, 1 kHz) pumped by a Nd:YLF laser (Spectra-Physics, Empower 15). The seed pulse was generated by a titanium sapphire laser (Spectra-Physics, Mai Tai VFSJW; fwhm 80 fs). The second harmonic generation of the fundamental light (400 nm, 3 μJ pulse-1) or the output of the optical parametric amplifier (780 nm, 3 μJ pulse-1, Spectra-Physics, OPA-800CF-1) was used as the excitation pulse. A white light continuum pulse, which was generated by focusing the residual of the fundamental light on a sapphire crystal after the computer controlled optical delay, was divided into two parts and used as the probe and the reference lights, of which the latter was used to compensate the laser fluctuation. The powder samples were dispersed into ethanol to be ink and then spread on cleaned glass cover slip. Both probe and reference lights were directed to the sample powder coated on the glass substrate, and the reflected lights were detected by a linear InGaAs array detector equipped with the polychromator (Solar, MS3504). The pump pulse was chopped by the mechanical chopper synchronized to one-half of the laser repetition rate, resulting in a pair of spectra with and without the pump, from which the absorption change (% absorption) induced by the pump pulse was estimated. The decay curves were fitted two-exponentially using the following equation:

$$y = y_0 + A_1\, exp\left(\frac{-(x - x_0)}{t_1}\right) + A_2\, exp\left(\frac{-(x - x_0)}{t_2}\right) \quad (3)$$

where, A_1 and A_2 represent the normalized amplitudes of each decay component and τ_1 and τ_2 are values of the lifetime components, respectively.

The average lifetime (τ_{av}) was calculated from the two lifetime components using the following expression:

$$\tau_{av} = \tau_1\, A_1\% + \tau_2\, A_2\% \quad (4)$$

Text S10. Single-particle photoluminescence (PL) spectroscopy.

C_3N_4 and C_3N_5 aqueous suspensions were dispersed in Milli-Q ultrapure water. The well-dispersed aqueous suspensions of samples were spin-coated on the cleaned quartz cover glass. The quartz cover glass was annealed at 100 °C for 1 h to immobilize the particles on the surface. Single-particle PL images and PL decay spectra were recorded by using an objective scanning confocal microscope system (PicoQuant, MicroTime 200) coupled with an Olympus IX71 inverted fluorescence microscope. The samples were excited through an oil-immersion objective lens (Olympus, UplanSApochromat, 100×, 1.4NA) acircular-polarized 405 nm picosecond laser controlled by aPDL-800B driver (PicoQuant). The emission from the sample was collected by the same objective and detected by a single photon avalanche photodiode (Micro Photon Devices, PDM 50CT) through a dichroic beam splitter (Chroma, 375rdc) and long pass filter (Chroma, HQ405CP).

Text S11. Electron paramagnetic resonance (EPR) measurement

Electron paramagnetic resonance (EPR, Bruker, EMX nano) technique at ambient temperature was used to explore free radicals and defective states. The samples were applied both ultrasonic force and light by a ultrasonic cleaner and a 300 W Xe lamp with a 420 nm cutoff filter. The *in situ* EPR measurement is studied by transferring generated sample to sample chamber of EPR with a peristaltic pump (**Figure S58**). All the samples were measured under the same conditions (Mod Amp: 1.000 G, Mod Freq: 100 kHz, Res Center: 125 mm, Res Length: 25 mm, Sweep time per sample: 30 s for offline samples and 2.5 s for samples *in situ* measurement; Sample interval: 0.5 s). The spectra were fitting by EPR software.

Text S12. DFT calculation methods and COMSOL simulation.

Theoretical simulations of dipole moments were performed with Gaussian 16 software package. Geometry-optimization was performed by adopting the B3LYP functional along with 6-31G(d) basis set. The most stable structure of the complex was determined after considering different alignments of carboplatin on C_3N_4 and C_3N_5. Calculations of frequency were accomplished at the parallel level of the theory (no

imaginary frequency) to confirm the optimized-geometries as true minima. Time-dependent TD-DFT with B3LYP/6-31G(d) was used to accomplish calculations for excited-state.

The first principle calculations were performed by the Vienna *ab initio* simulation package (VASP) using the generalized gradient approximation (GGA) and Perdew-Burke-Ernzerhof (PBE) method base on density functional theory (DFT).[5] The projector augmented wave (PAW) was used to describe ions and electron interaction. The kinetic energy cutoff was set at 450 eV. C_3N_4 and C_3N_5 were employed as the models to further determine the reasons for the excellent catalytic activity. The cell parameters of C_3N_4 and C_3N_5 are a=14.27400 Å, b=14.23270 Å, c=8.46310 Å and a=15.03930 Å, b=15.03930 Å, c=25.00000 Å, respectively. In the vertical direction, a vacuum layer of about 15 Å in thickness was introduced to avoid the interaction between neighboring image structures.

In all the calculations, we use 3×2×1 for the Monkhorst-Pack k-point for periodic crystal structure and surface model. The convergence threshold for energy was set at 10^{-5} eV.[6] The equilibrium lattice constants were optimized with maximum stress on each atom within 0.05 eV/Å.

The adsorption energy (ΔE_{ads}) were defined as follows：

$$E_{ads} = E_{ad/sub} - E_{ad} - E_{sub} \quad (5)$$

where $E_{ad/sub}$, E_{ad} and E_{sub} are the optimized adsorbate/substrate system, the adsorbate in the structure and the clean substrate respectively. Usually, a more negative E_{ads} value reflects a stronger adsorption.

The free energy was calculated using the equation:

$$\Delta G = \Delta E + \Delta ZPE - \Delta TS \quad (6)$$

where ΔG, ΔE, ΔZPE, and TS represented the free energy, total energy from DFT calculations, zero-point energy, and entropic contributions (T was set to be 300 K), respectively.

The piezoelectric potential distribution on the C_3N_4 and C_3N_5 nanosheet were simulated by COMSOL Multiphysics Software. According to the previous reported

method,[7] 2D geometry parameter was 100 nm × 100 nm × 20 nm, where the same domain was uniformly distributed for the simulations. For the boundary settings, the upper end of the cantilever was taken to be free in response to the applied force.

The applied pressure of all variable pressure simulations is 2 GPa. According to previous report, the sound pressure fluctuates in a form of wave during the propagation of ultrasonic waves in a fluid.[8, 9] Water molecules go through a process of ultrasonic cavitation where they can be squeezed out in the positive phase of the pressure (compression) and then disintegrated in the negative phase (decompression). Such an alternate cyclic-procedure results in the formation and growth of cavitation bubbles, which is able to release a huge impact force as high as 0.1–2 GPa when the cavitation bubbles implode at a critical size.[10] In particular, the total pressure (P) in a cavitation bubble is derived from following equation:[10]

$$P = p_0 + 2\sigma/R_0 \quad (7)$$

where p_0 is the disturbed pressure, σ is the surface tension and R_0 is the initial bubble radius. Mukasa et al. reported that the released pressure inside a bubble in n-dodecane can reach up to 2 GPa at the acoustic frequency of 19.5 kHz and acoustic pressure amplitude of 1.3 atm under the calculating conditions that temperature (T_0) = 298 K, p_0 = 1 atm, and R_0 = 50 μm.[10] Based on these initial findings, the ultrasound condition offered by our experiment (frequency of 40 kHz and power of 100 W) can generate pressure of up to 2 GPa, and 2 GPa was chosen for theoretical simulation of variable pressure.

Supplementary Figures

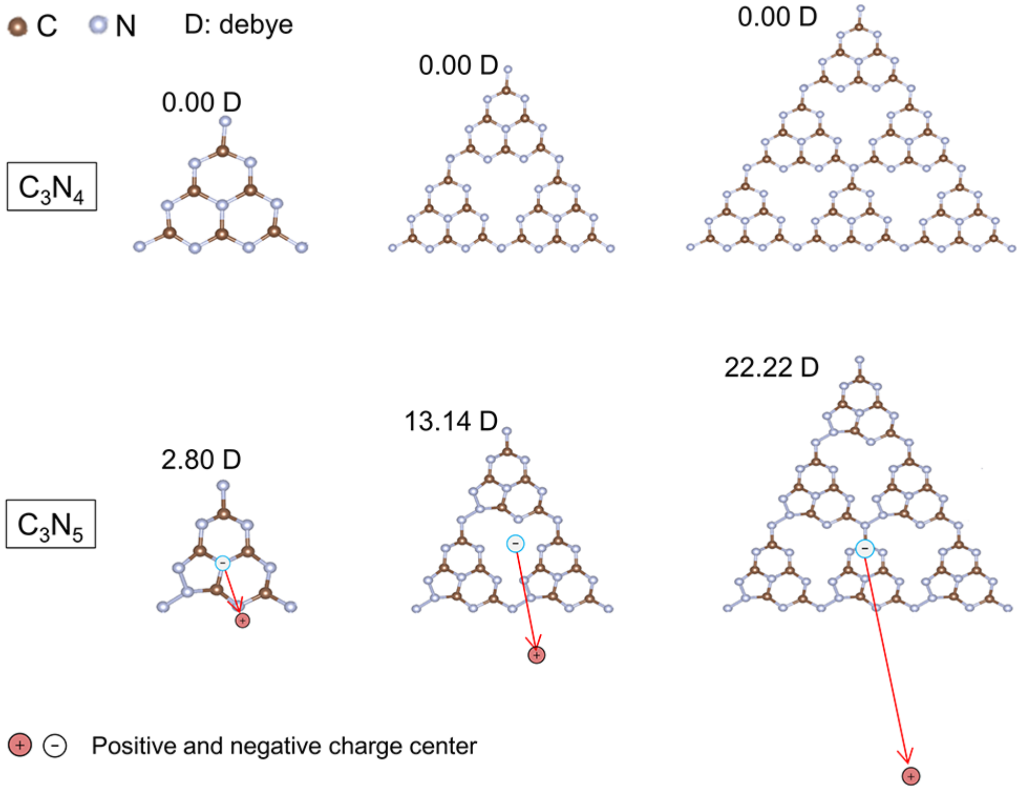

Figure S1: The dipole moments of C_3N_4 and C_3N_5 with different structural unit numbers.

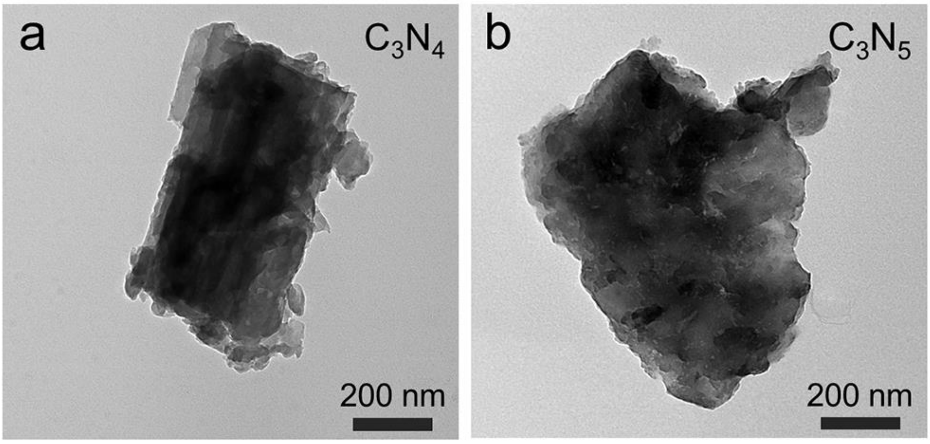

Figure S2: TEM images of C_3N_4 (**a**) and C_3N_5 nanosheets (**b**).

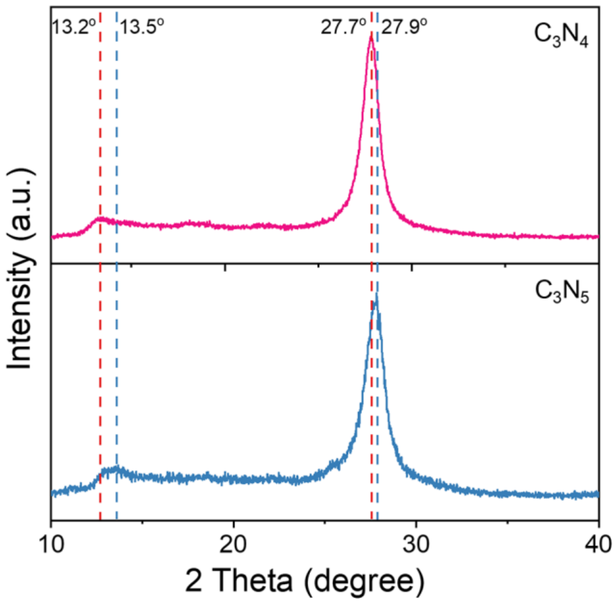

Figure S3: XRD patterns of C_3N_4 and C_3N_5.

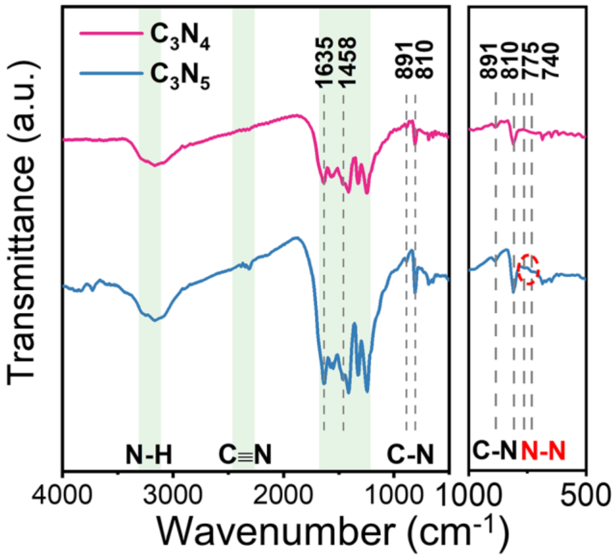

Figure S4: FT-IR spectra of C_3N_4 and C_3N_5.

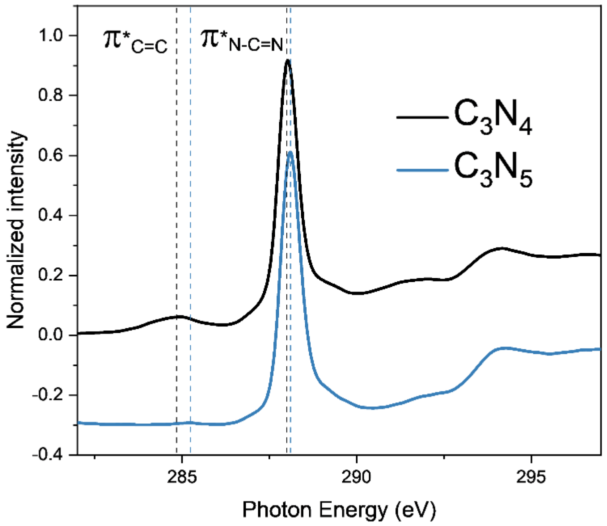

Figure S5: C K-edge and N K-edge NEXAFS spectra of C_3N_4 and C_3N_5.

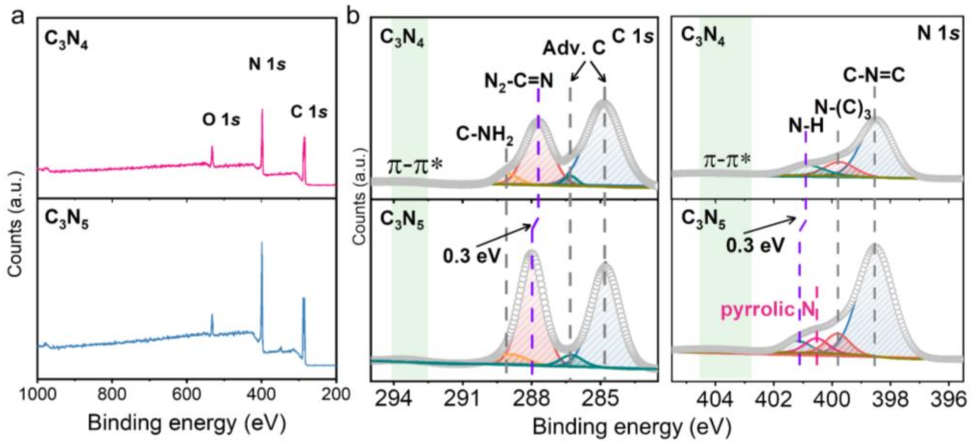

Figure S6: XPS spectra of C_3N_4 and C_3N_5. a, Survey XPS spectra. **b,** High-resolution XPS spectra of C 1s and N 1s.

Note: The survey X-ray photoelectron spectroscopy (XPS) spectra are shown in **Figure S6a**, which displays that the prepared C_3N_5 is composed of C and N. The small amount oxygen is due to the adsorbed CO_2 or H_2O on the material surface. The C 1s XPS spectrum for C_3N_5 (**Figure S6b**) shows a sharp peak at 287.9 eV (assigned to the N_2-C=N group), which displays a slight shift of 0.3 eV relative to C_3N_4. These results indicate that C_3N_5 has a stronger electronic coupling for the C-N or C=N bond than C_3N_4. This is in accordance with the introduced triazole moieties, which raise the number of N atoms and thus enhance the electron-absorption ability of C atoms. The peaks at

285.7 and 284.8 eV are attributed to adventitious C (Adv. C),[11] while another peak at 289.1 eV is ascribed to the C-NH_2 group. The N 1s XPS spectrum (**Figure S6b**) of C_3N_4 shows three peaks centered at 398.5, 399.8, and 400.9 eV, corresponding to C-N=C, N-$(C)_3$, and N-H, respectively.[12] Interestingly, there is also a sharp peak located at 400.6 eV for C_3N_5, which corresponds to the pyrrolic nitrogen sourced from the triazole group,[13, 14] and the N-H peak of C_3N_5 displays a slight shift to a high binding energy relative to C_3N_4 (by 0.3 eV) owing to the formation of cyanamide groups.[15] The relative percentages (at%) of different peaks in the C 1s and N 1s XPS spectra are displayed in **Tables S1** and **S2**.

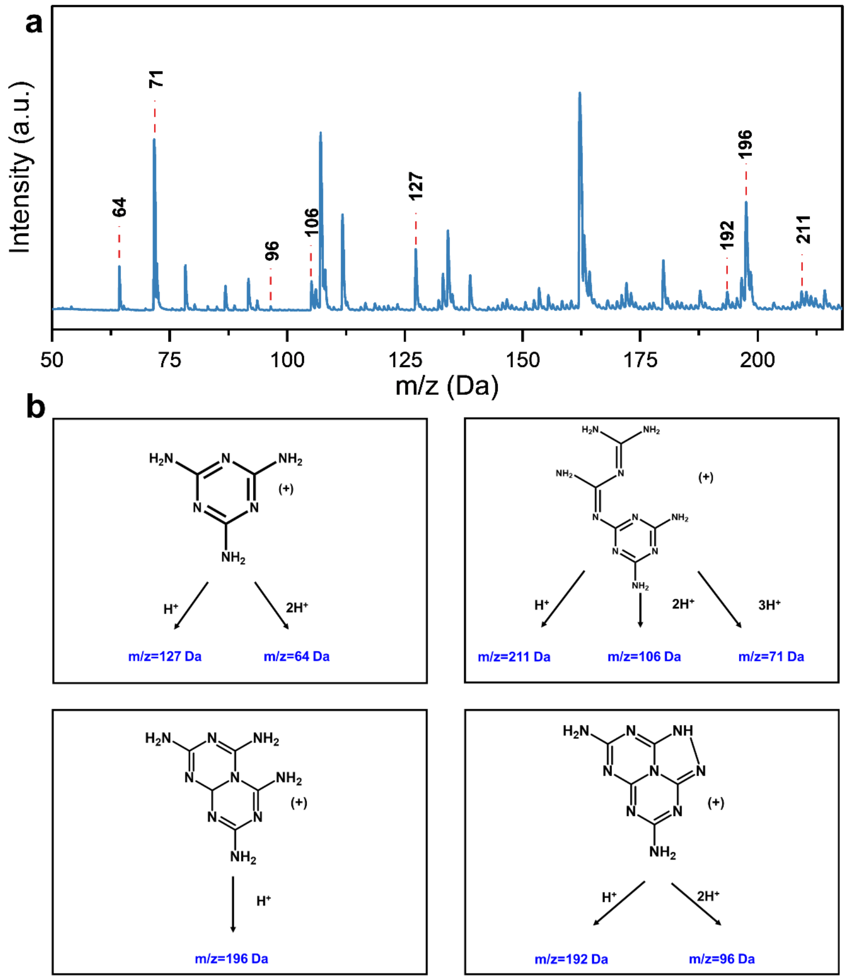

Figure S7: MALDI-TOF-MS spectra (a) of CN-400°C; Major species (b) constituting the precipitate formed from CN-400°C. The solid sample was adopted for testing.

Note: MALDI-TOF-MS was used to determine the chemical structure of intermediates.

Figure S7a presents the whole MALDI-TOF-MS spectra of the intermediate of CN prepared at 400°C. Since the amino groups on the carbon nitride are easily charged with positive protons, we tested the intermediates in positive ion mode. The peaks with m/z of 64, 71, 96, 106, 127,192, 196 and 211 Da are observed in the MALDI-TOF MS spectra of CN-400. Therefore, some possible molecular structures of the intermediates in the CN polymerization are listed in **Figure S7b**. Based on this, the above m/z can correspond to the eight positively ionized products of the four molecules as above. This implies the possible existence of these four intermediates. Other excess peak may be due to the effect of incomplete polymerization of 3-AT and other impurities.

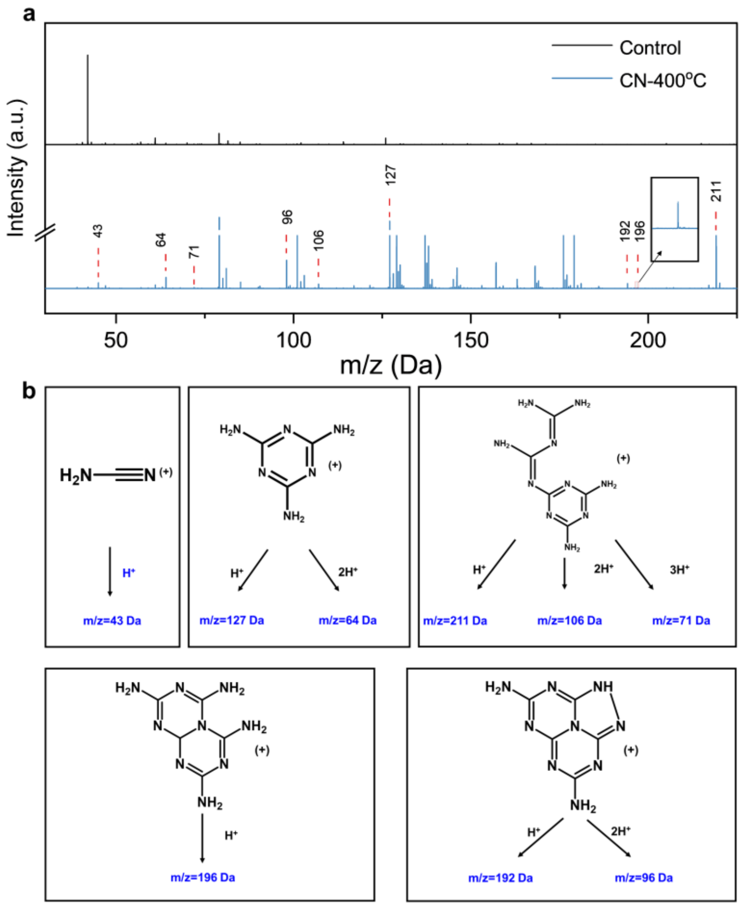

Figure S8: LC-TOF-MS spectra (a) of H_2O and CN-400°C; Major species (b) constituting the precipitate formed from CN-400°C**.** DMSO was used as a solvent for CN-400°C, and DMSO was used as a control sample.

Note: LC-TOF-MS was used to determine the chemical structure of intermediates.

Figure S8a presents the whole LC-TOF-MS spectra of the intermediate of CN prepared at 400°C. We also tested the intermediates in positive ion mode, because the amino groups on the carbon nitride are easily charged with positive protons. The peaks with m/z of 43, 64, 71, 96, 106, 127,192, 196 and 211 Da are observed in the LC-TOF-MS spectra of CN-400ºC. Therefore, the positively ionization mode of these possible intermediates is shown in are listed in **Figure S8b**. These results are also very consistent with the results of MALDI-TOF-MS. This also demonstrates the possible existence of these four intermediates.

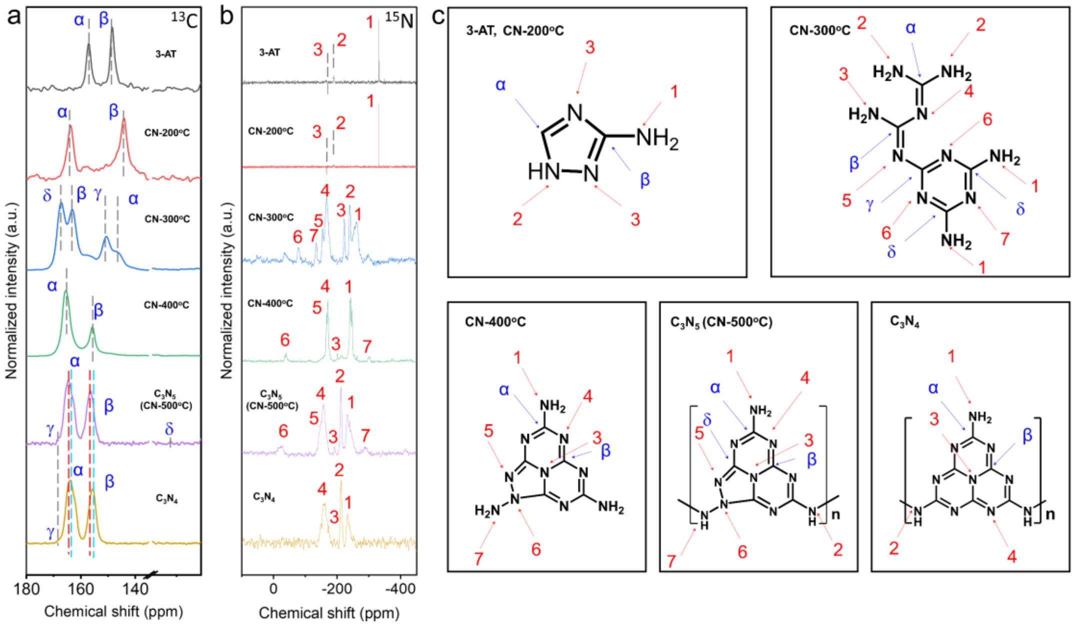

Figure S9: ^{13}C and ^{15}N NMR spectra (**a** and **b**) of 3-AT, CN-200ºC, CN-300ºC, CN-400ºC, C_3N_5 (CN-500ºC) and C_3N_4 and possible structural model representation (**c**).

Note: The chemical structure of the intermediates was further demonstrated by NMR spectroscopy (**Figure S9**). The ^{13}C NMR spectra (**Figure S9a**) of 3-AT display two peaks at 157 and 148 ppm, attributed to N-C-NH (α) and N_2-C-NH_2 (β) of 3-AT, respectively, which was also proved by simulated ^{13}C NMR spectra (**Figure S10**). CN-200ºC also exhibits two peaks at 163.5 and 144.1 ppm, attributed to two C atoms of 3-AT, respectively, which may be due to the fact that CN-200ºC does not change the chemical structure of 3-AT because of the boiling point of 3-AT is 244.9°C, but high temperature heating for 3-AT affects the chemical shifts of the elements. Similarly, the ^{15}N NMR

spectra of both 3-AT and CN-200^oC display three peaks at -331, -188 and -170 ppm, attributed to C-NH_2 (N1), C-NH-N (N2) and C-N-C(N) (N3) in 3-AT, respectively (**Figure S9b**). The ^{13}C NMR spectra (**Figure S9a**) of CN-300°C display four peaks at 167, 163, 151 and 146 ppm, attributed to N_2-C-NH_2 (δ), NH_x-C-N_2 (β), CN_3 (γ) and $(NH_2)_2$-C-N (α), respectively, which was also proved by simulated ^{13}C NMR spectra (**Figure S10**); while the ^{15}N NMR spectra (**Figure S9b**) of CN-300°C display seven peaks at -74.5, -133.8, -154.4, -166, -220.4, -237.6 and -261.1 ppm, attributed to N atoms in seven different chemical environments, respectively. The ^{13}C NMR spectra of three samples (CN-400^oC, C_3N_5 and C_3N_4) display two peaks at around 165 and 156 ppm, attributed to C_2N-NH_x (α) and C_3N (β) in the heptazine units in C_3N_5, respectively.[16] Notably, the peak of C_3N_5 is slightly shifted relative to C_3N_4 due to the triazole group in C_3N_5. Compared to C_3N_4, CN-400^oC and C_3N_5 (CN-500°C) showed significant increases at 158ppm, due to the inability of NMR to discern the hidden peak in C_3N_5, which was also proved by simulated ^{13}C NMR spectra. The ^{15}N NMR spectrum of C_3N_4 displays four signals at -156.6, -191.6, -211.6 and -227.6 ppm, assigned to NC_2 (N4), central NC_3 (N3), bridged NH (N2) and NH_2 (N1), respectively.[16] Similar to C_3N_4, the ^{15}N NMR spectrum of CN-400^oC and C_3N_5 (CN-500°C) exhibits similar peaks, and some new signals at -22.4, -146.7 and -288.8 ppm are observed. These new signals are attributed to C-N (N6)-N_2, C=N (N5)- N and N-NH (N7) from the triazole group in C_3N_5, respectively, due to the strong nitrogen-proton coupling with neighboring ammonia groups.[17, 18] Interestingly, almost no a signal of bridged NH (N2) was observed in the ^{15}N NMR spectrum of CN-400^oC, which may be due to the formation of a single structural unit of C_3N_5 at 400^oC. All these characterizations fully indicate that nitrogen-rich carbon nitride with triazole and two triazine groups in C_3N_5 was synthesized.

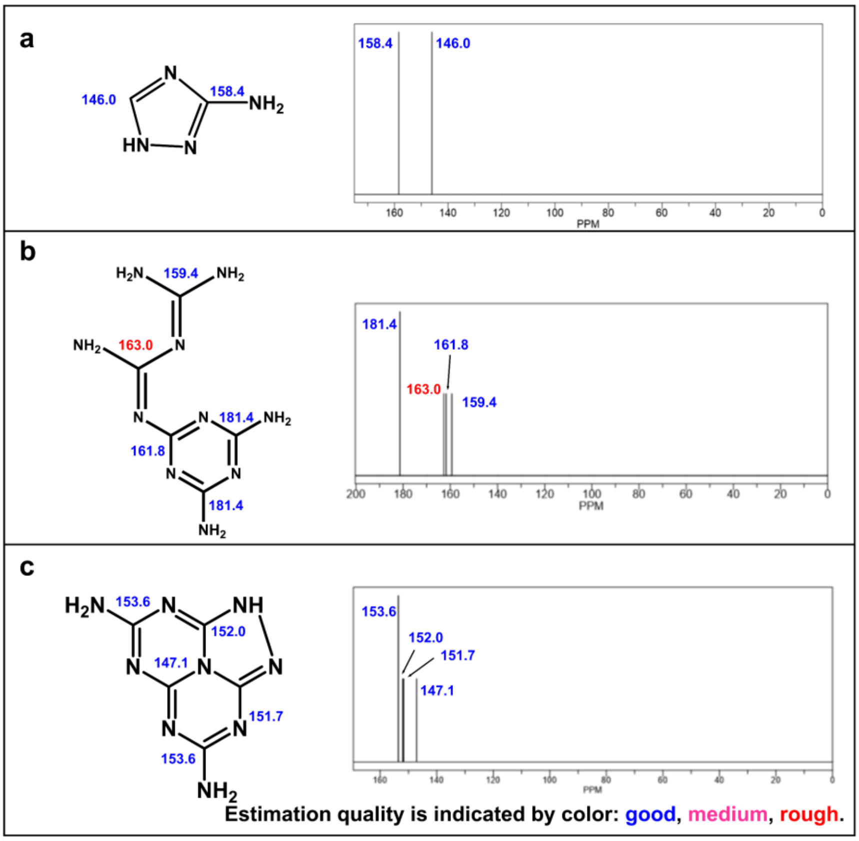

Figure S10: Structural model and theoretical correspondence of ^{13}C NMR chemical shift produced from ChemDraw.

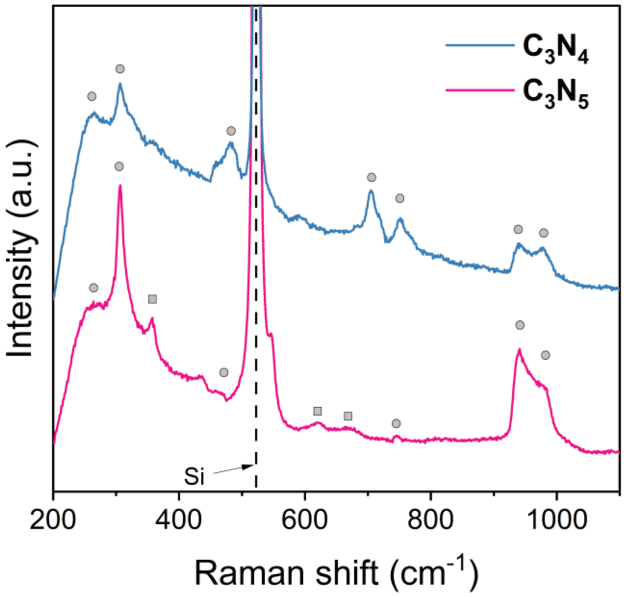

Figure S11: Micro Raman spectra of C_3N_4 and C_3N_5. The circles and squares indicate Raman modes of triazine and triazole rings, respectively. 524 cm^{-1} shows the signal of

Si, since the substrate is made of silicon.

Note: The characteristic Raman peaks of g-C_3N_4 appear at 260, 316, 472, 715, 753, 980, 1233 and 1562 cm^{-1}. These peaks are assigned to breathing modes of the triazine ring.[19] On the other hand, triazole-based C_3N_5 show additional vibration modes at 362, 618 and 667 cm^{-1} corresponding to breathing modes of the triazole ring.[20]

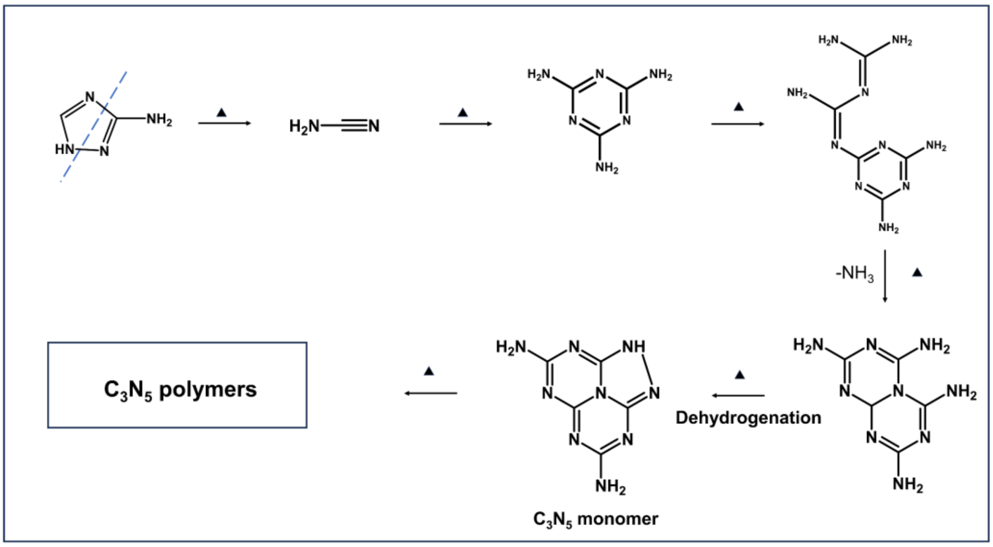

Figure S12: The possible synthetic steps of C_3N_5.

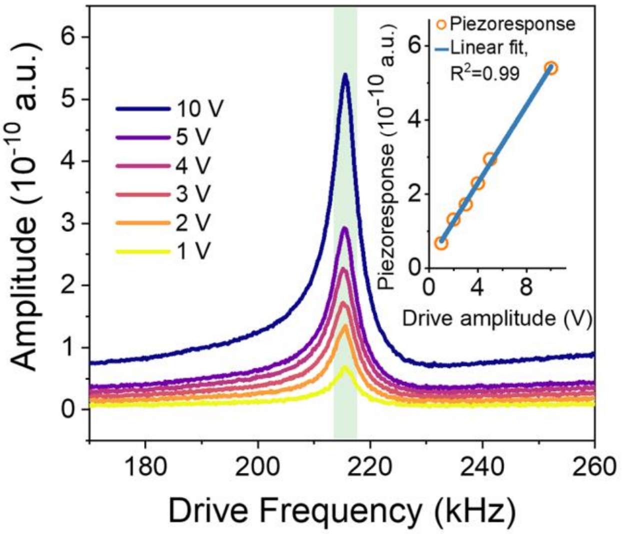

Figure S13: PFM resonant responses for different applied voltages. The inset shows linear relationship between piezo response and drive frequency.

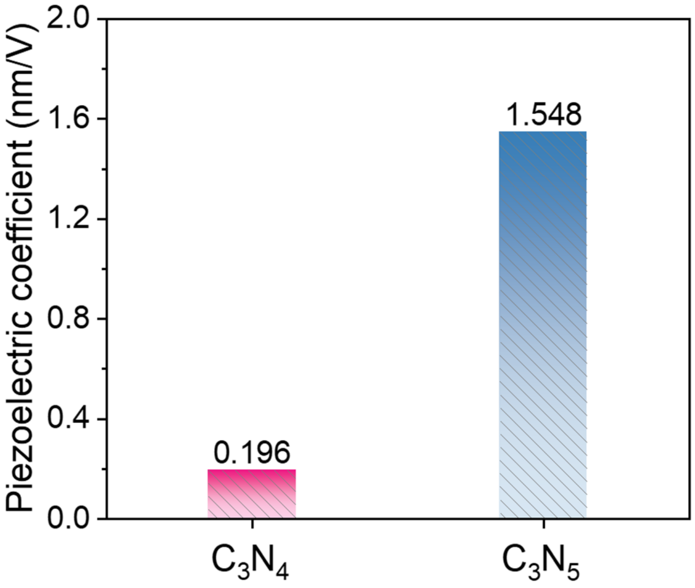

Figure S14: The piezoelectric coefficient of C_3N_4 and C_3N_5.

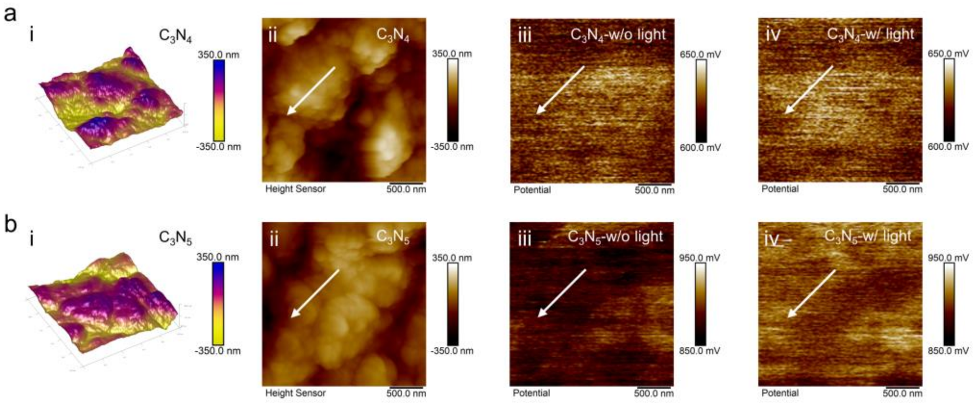

Figure S15: Surface morphologies and corresponding KPFM potential images of C_3N_4 (a) and C_3N_5 (b). i, AFM 3D topography images. ii, AFM 2D topography images. iii, iv, contact potential difference (CPD) of C_3N_4/C_3N_5 with and without light. The arrow is the selected surface potential area.

Note: Figures S15 a-b-i,ii present the topography images of all samples with irregular shapes.

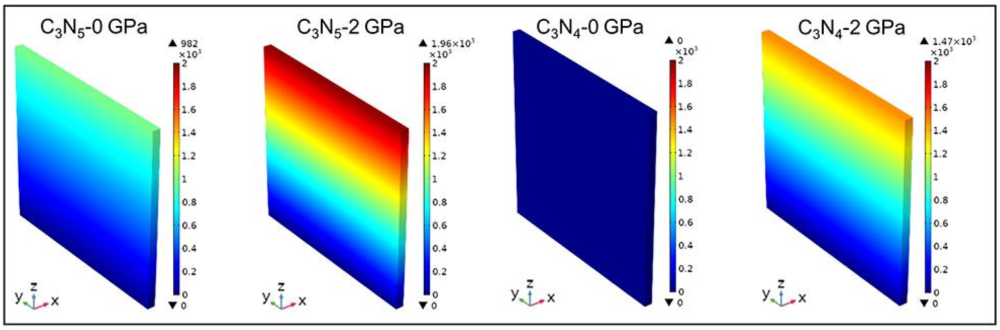

Figure S16: COMSOL simulation for piezoelectric potential distribution of C_3N_4 and C_3N_5 with 0 and 2 GPa.

Notes: As shown in **Figure S16**, without applied pressure, intrinsic C_3N_5 presents an inhomogeneous piezoelectric potential distribution relative to C_3N_4 due to the introduction of triazole groups in C_3N_5. While applying an extra pressure field of 2 G Pa, C_3N_5 shows strongest piezoelectric potential distribution, and they follow this order: C_3N_5-2G Pa (1.96×10^3 mV) > C_3N_4-2G Pa (1.47×10^3 mV) > C_3N_5-0G Pa (0.98×10^3 mV) > C_3N_4-0G Pa (0 mV).

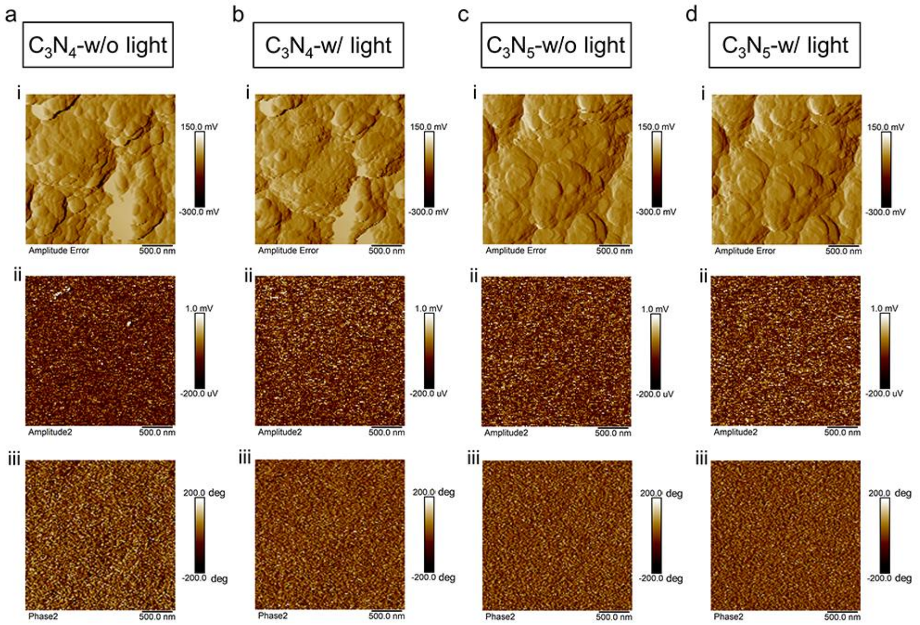

Figure S17: PFM analysis. **a**, C_3N_4 w/o light. **b,** C_3N_4 w/ light. **c,** C_3N_5 w/o light. **d,** C_3N_5 w/ light. **i,** amplitude error image. **ii,** amplitude image. **iii,** phase image.

Note: In the amplitude and phase images of samples, there are different colors and strengths contrast, representing different directions of piezoelectric polarization.

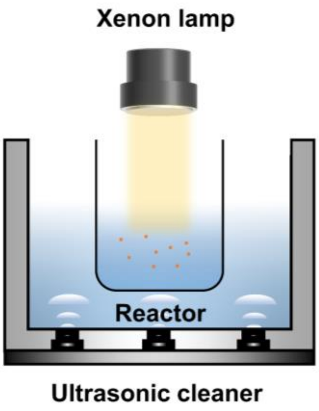

Figure S18: System for H_2O_2 photosynthesis with Us condition.

Notes: Figure S18 displays the photocatalytic H_2O_2 production with Us system. It contains a Xenon lamp source, an ultrasonic cleaner and a glass reactor.

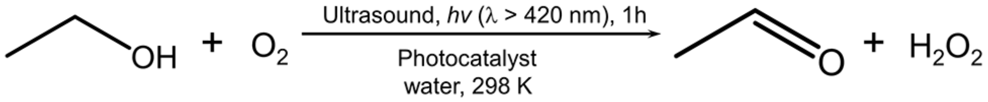

Figure S19: Photocatalytic reaction scheme for H_2O_2 production with EtOH aerobic oxidation. Experimental conditions: catalyst (0.5 g L^{-1}) with 10 vol% EtOH under Us and Vis light illumination (λ ≥ 420 nm), T = 25°C, water.

Note: The photocatalytic production with Us of H_2O_2 from the resultant samples was tested in the presence of ethanol (EtOH) (**Figure S19**).

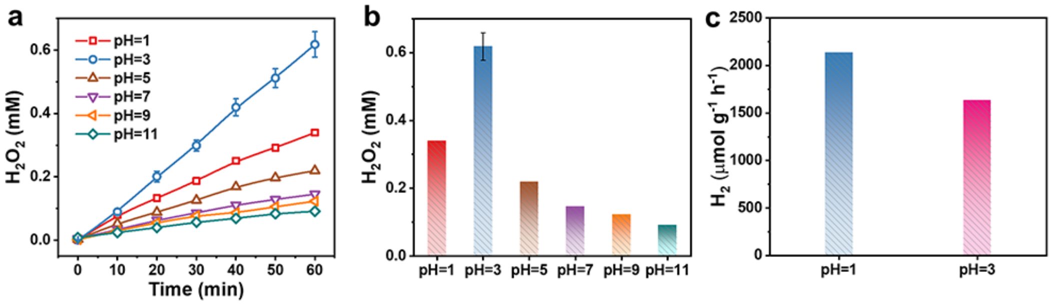

Figure S20: a, Time profiles of photocatalytic H_2O_2 production by C_3N_5 under Us over a wide pH range (pH 1–11). **b**, The corresponding histograms of H_2O_2 yield at 60min. **c**, H_2 yield at 60min over pH=1 and 3.

Note: H_2O_2 at millimolar level was produced over C_3N_5 over a wide pH range (pH 1–11), with higher yields obtained at lower pH (down to pH 3.0) due to elevated proton concentrations in acidic media (**Figures S20a** and **b**). However, the H_2O_2 production activity significantly decreased when the pH was further lowered from pH=3 to pH=1, which indicates the occurrence of the competitive reaction of H_2 evolution ($2H^+ + 2e^- \rightarrow H_2$ (0 V $vs.$ NHE)) along with the production of H_2O_2 (**Figure S20c**).

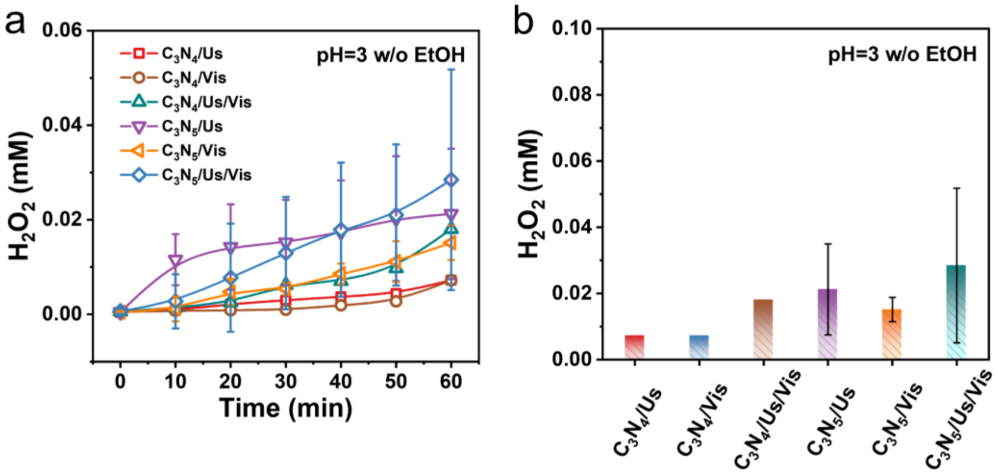

Figure S21: a, Time profiles of H_2O_2 production by C_3N_4 and C_3N_5 in various scenarios (pH=3 w/o EtOH). **b**, The corresponding histograms of H_2O_2 yield at 60min.

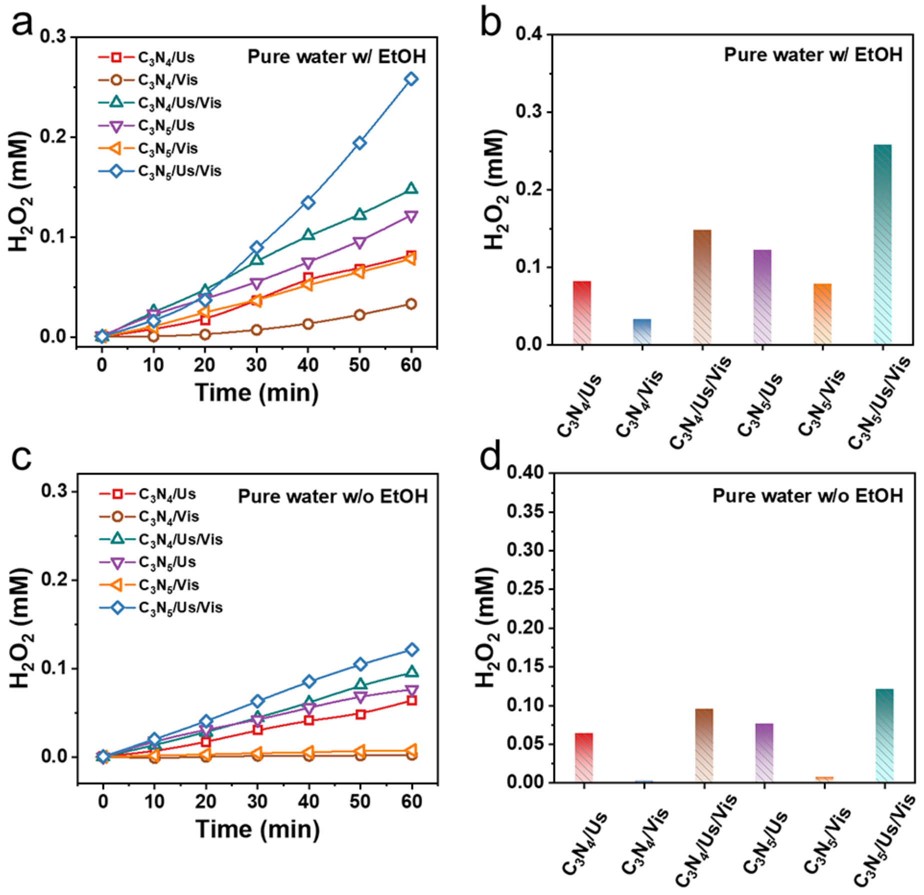

Figure S22: **a** and **c**, Time profiles of H_2O_2 production by C_3N_4 and C_3N_5 in various scenarios (pure water w/ and w/o EtOH). **b** and **d**, The corresponding histograms of H_2O_2 yield at 60min.

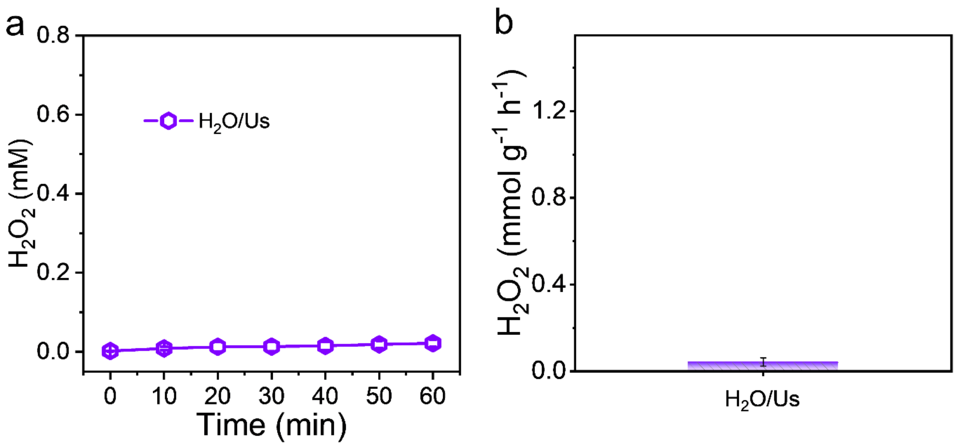

Figure S23: **a**, Time profiles of H_2O_2 production via pure water with Us. **b**, Corresponding histograms of the H_2O_2 yield at 60 min.

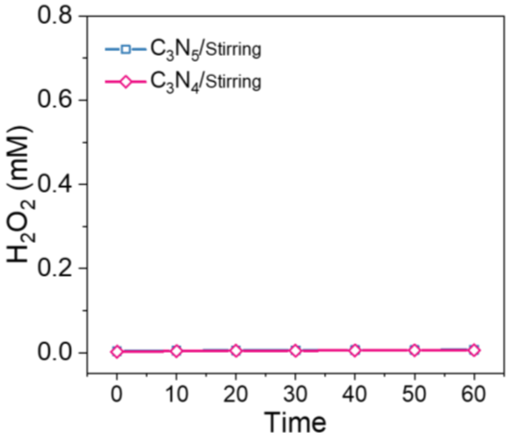

Figure S24: Time profiles of H_2O_2 production by stirring-only with C_3N_4 and C_3N_5.

Notes: A separate stirring experiment without light was added as a control. In **Figure S24**, C_3N_4 and C_3N_5 produce almost no H_2O_2 with only stirring.

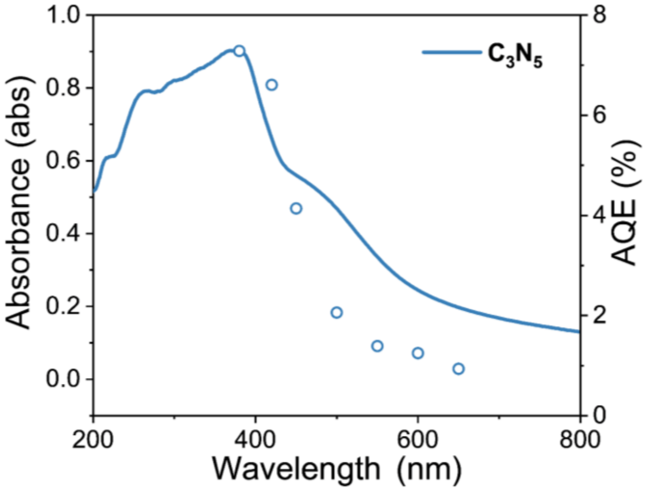

Figure S25: Apparent quantum yield (AQY) of H_2O_2 production at specific wavelengths superimposed with its UV-Vis absorption curve.

Note: The apparent quantum efficiency (AQE) of C_3N_5 was calculated at specific wavelengths and shown to approximately match with the UV-Vis spectrum (**Figure S25**). C_3N_5 exhibited an apparent quantum yield (AQY) of H_2O_2 production close to 7.3%, 6.7%, 4.1%, 2.1%, 1.4%, 1.2% and 0.9% at wavelengths of 380 nm, 420 nm, 450 nm, 500 nm, 550 nm, 600 nm and 650 nm, respectively.

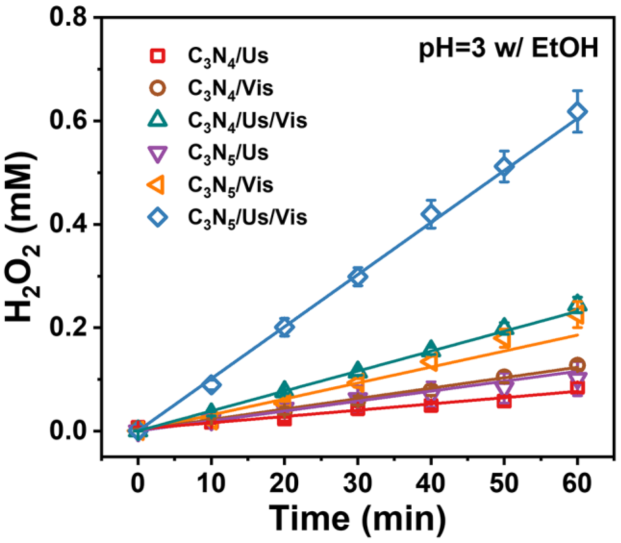

Figure S26: The first-order kinetics model of C_3N_4 and C_3N_5 for H_2O_2 production in various scenarios.

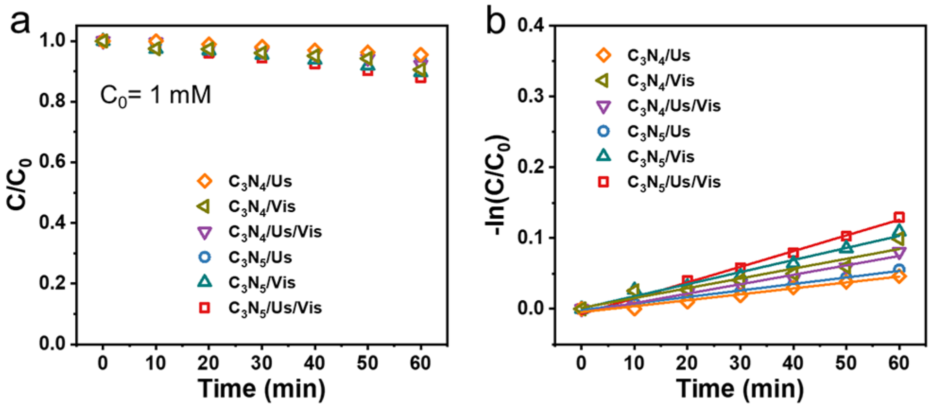

Figure S27: a, The decomposition of H_2O_2 (1 mM) by C_3N_4 and C_3N_5 in various scenarios. **b**, The zero-order kinetics model.

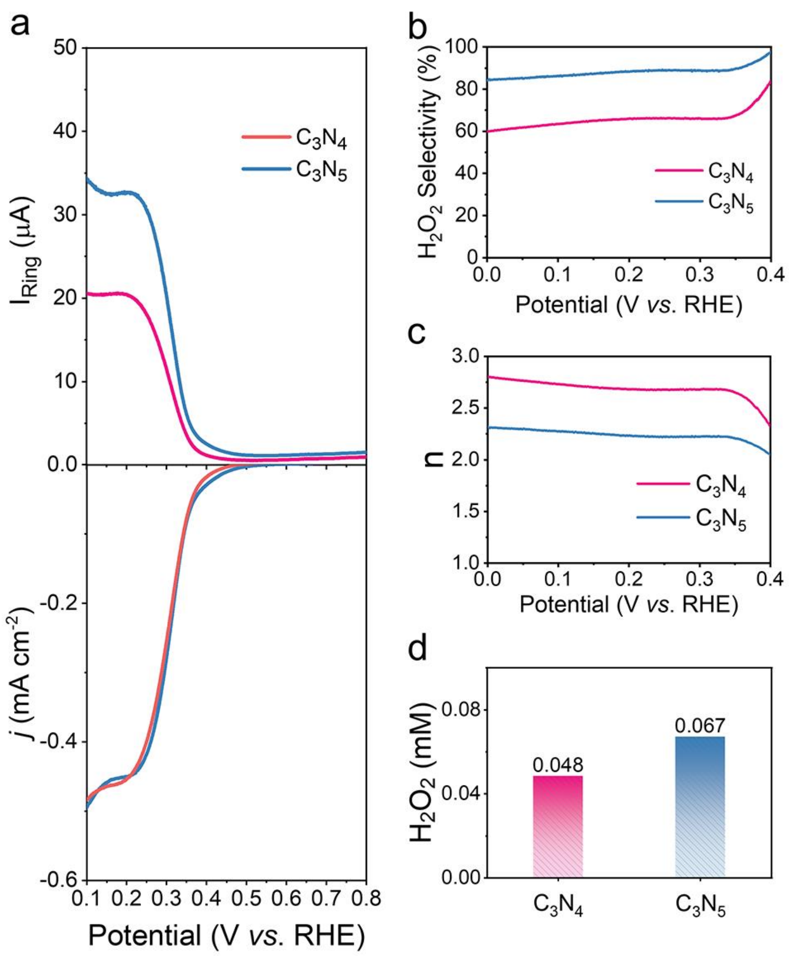

Figure S28: The electrochemical production of H_2O_2. a, ORR polarization curves. **b**, H_2O_2 selectivity. **c**, the corresponding number of transferred electrons. **d**, H_2O_2 produced by ORR after 5 min in the RRDE reactor over C_3N_4 and C_3N_5.

Notes: The electron transfer pathway was evaluated by rotating ring-disk electrode (RRDE) analysis at 1600 rpm in O_2 saturated phosphate buffer solution (0.1 M, pH 7).[21, 22] The ORR polarization curves are displayed in **Figure S28**. Compare with C_3N_4, C_3N_5 shows the higher onset potential (0.51 V *vs*. 0.45 V), and significant higher ring current (**Figure S28a**). A high H_2O_2 yield with 92% selectivity at 0.31 V (*vs*. RHE) is observed in C_3N_5 (**Figure S28b**). Besides, in contrast to C_3N_4, C_3N_5 shows approximately two-electron ($2e^-$) transfer (**Figure S28c**) from 0.0 to 0.4 V (*vs*. RHE). The electrochemical H_2O_2 yield of C_3N_5 at 10 min was higher than that of C_3N_4 (**Figure S28d**). These results indicate triazole-based C_3N_5 has outstanding $2e^-$ ORR for H_2O_2 production with high selectivity.

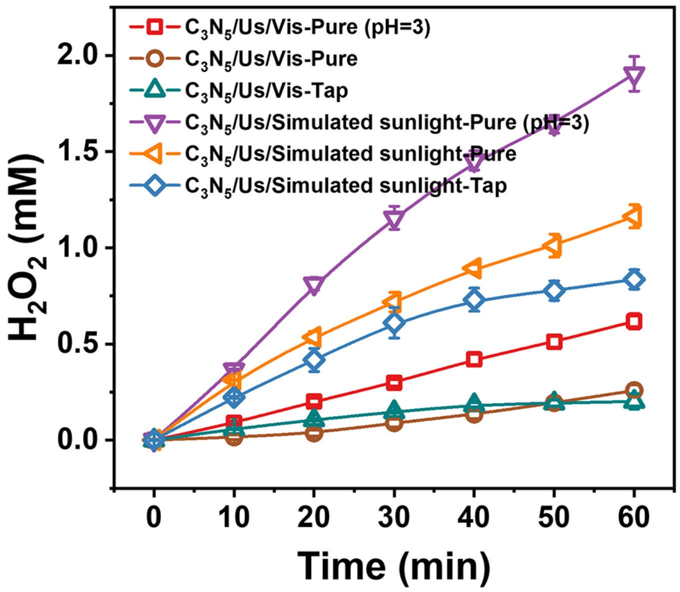

Figure S29: Time profiles of photocatalytic H_2O_2 production with Us over C_3N_5 in pH=3, DI and tap water under visible light (λ ≥ 420 nm) and simulated sunlight.

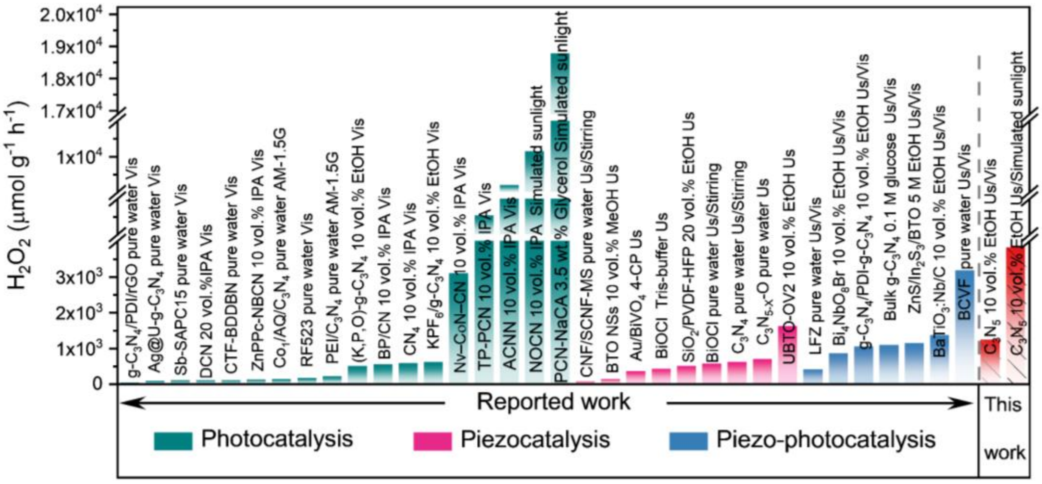

Figure S30: H_2O_2 production rates for C_3N_5 in this work compared with reported work. Corresponding reports are shown in **Table S4**.

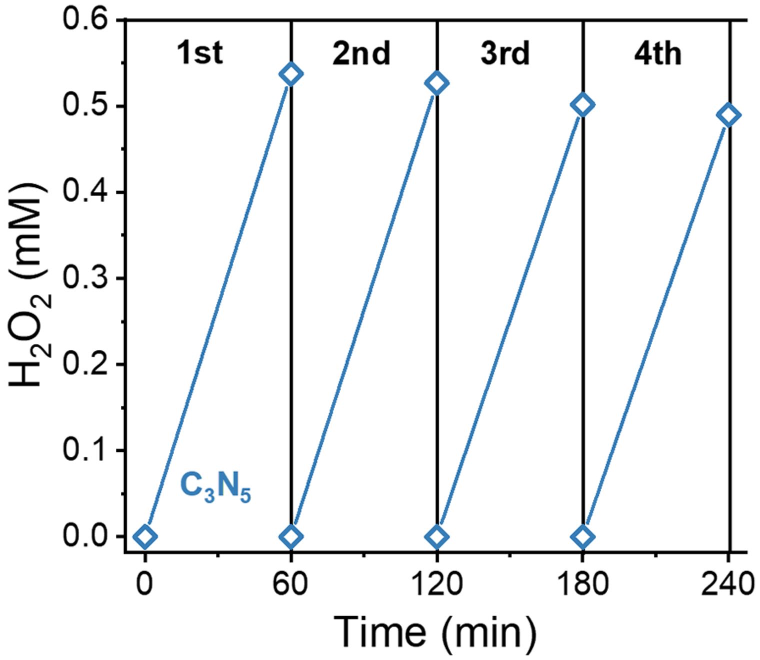

Figure S31: Repeated photocatalytic cycles of H_2O_2 production with Us of C_3N_5 with both Us and Vis light irradiation (pH = 3) containing 10 vol% EtOH.

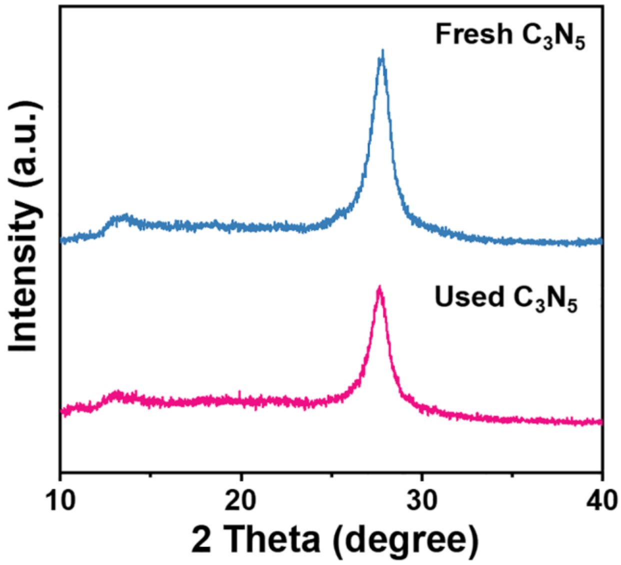

Figure S32: XRD patterns of fresh and used C_3N_5.

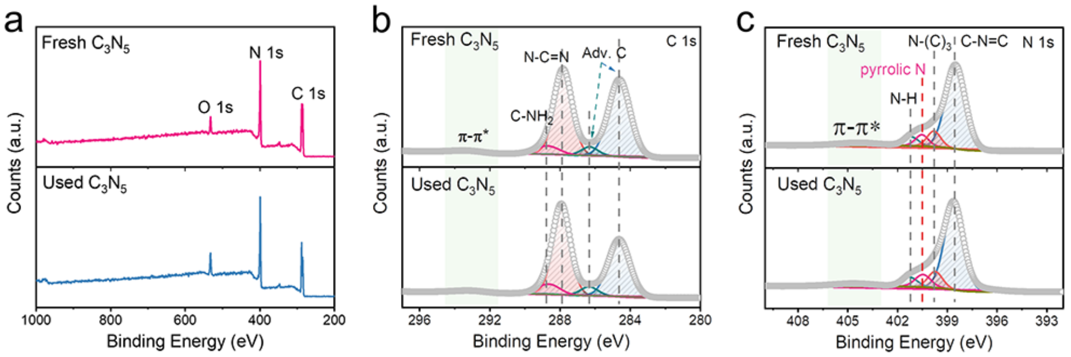

Figure S33: XPS spectra of fresh and used C_3N_5. **a**, Survey XPS spectra. **b,c**, High-resolution C 1s and N 1s.

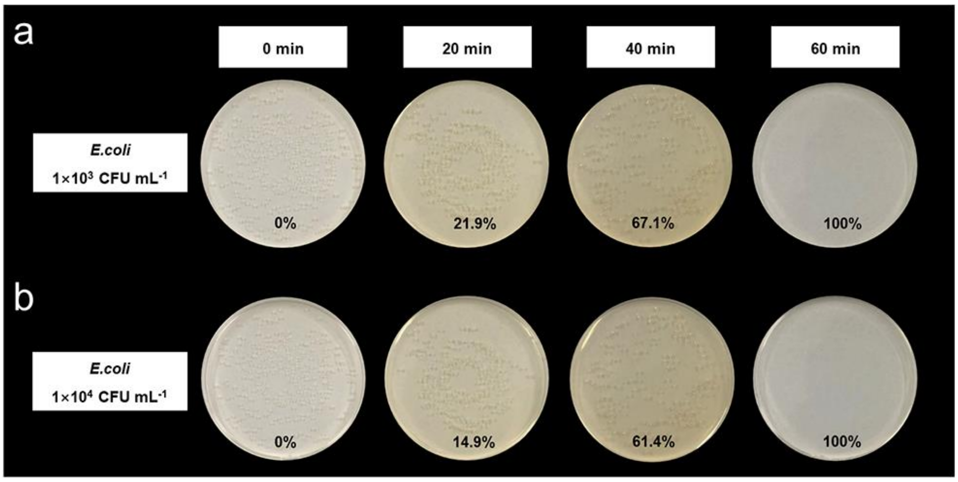

Figure S34: The disinfection of *E. coli* with different initial concentrations (**a** and **b**) using the H_2O_2 generated from the photocatalytic ORR reaction over C_3N_5 with Us. The growth inhibition in % is indicated in each figure.

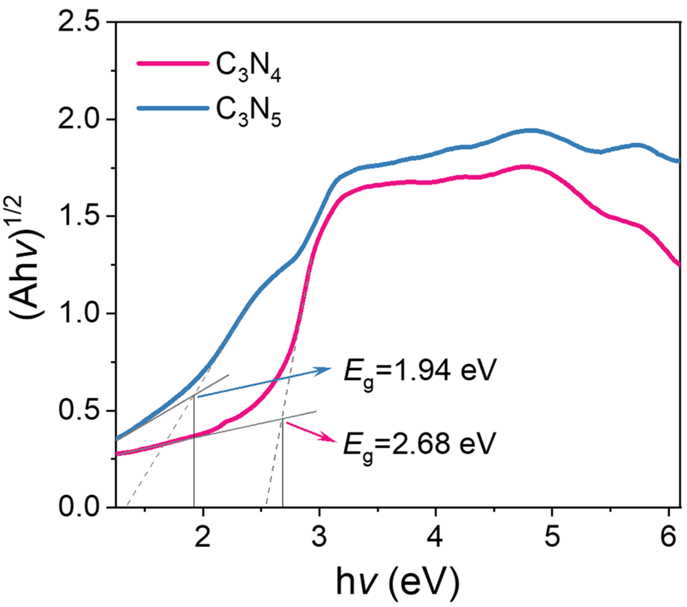

Figure S35: Tauc plots of C_3N_4 and C_3N_5.

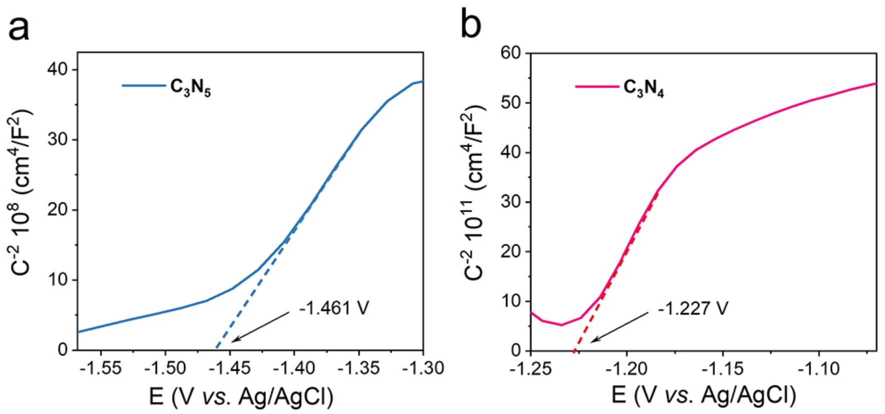

Figure S36: Mott-Schottky plots of C_3N_5 (**a**) and C_3N_4 (**b**).

Notes: Tests of Mott-Schottky plots are investigated to obtain specific band position of C_3N_5 and C_3N_4. The positive straight line slope under constant frequency of 1000 Hz manifests that C_3N_5 and C_3N_4 are n-type semiconductor.[1] Additionally, the flat band potential (V_{fb}) can be estimated by extrapolating the line to $1/C^2 = 0$ with the following equation:

$$\frac{1}{C^2}=\frac{2}{A^2 e \varepsilon_r \varepsilon_{0} N_d}\left(V\text{-}V_{fb}-\frac{kT}{e}\right) \quad (8)$$

where *C* is the specific capacity, *A* is the effective surface area, ε_r and ε_0 are dielectric constants of the catalyst and vacuum, respectively, *e* is elementary charge and N_d is

the carrier concentration of the catalyst, V is the applied potential while V_{fb} is the flat band potential, k is the Boltzmann constant and T represents absolute temperature.[23] Generally, conduction band (CB) of n-type semiconductor is more negative (ca. 0.2 V) than Fermi level which is same as flat band potential, while $E^{\theta}_{Ag/AgCl}$ = 0.198 V (vs. RHE).[24] As show in **Figure S36**, the flat band potentials of C_3N_5 and C_3N_4 are -1.461 and -1.227 V (*vs*. Ag/AgCl), respectively. Hence, their CB edge (*vs*. RHE) can be expressed as:

$$E_{CB}\ (C_3N_5) = -1.461 + 0.198 - 0.2 = -1.463\ V \quad (9)$$

$$E_{CB}\ (C_3N_4) = -1.227 + 0.198 - 0.2 = -1.229\ V \quad (10)$$

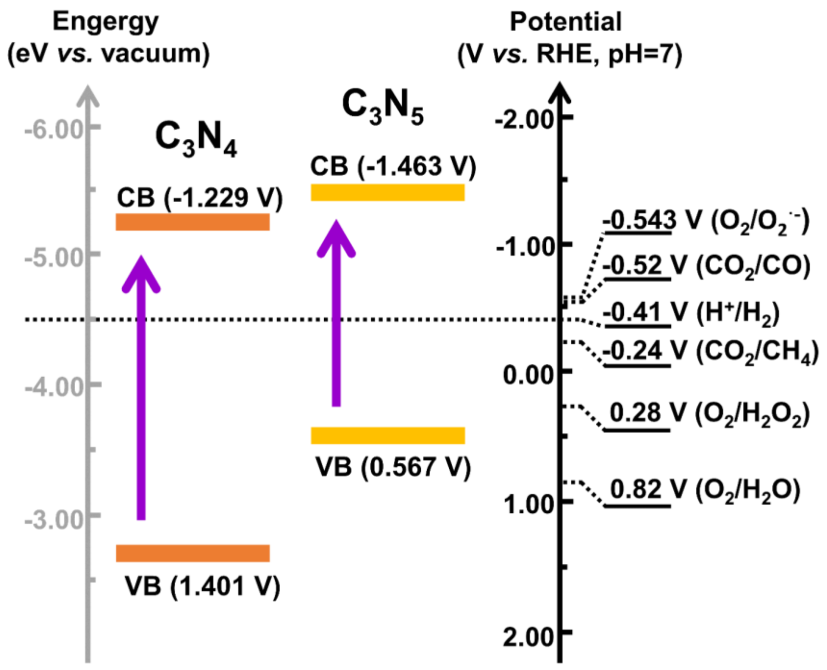

Figure S37: Energy band structures of C_3N_4 and C_3N_5.

Note: In contrast to conventional C_3N_4, C_3N_5 exhibits a wider visible (Vis) light absorption capacity and a narrower bandgap, suggesting easier photoexcitation performance, and a more negative reduction potential for easier photocatalytic ORR activity. These fully confirm that C_3N_5 has excellent Vis light utilization ability and the prospect of photocatalytic H_2O_2 production.

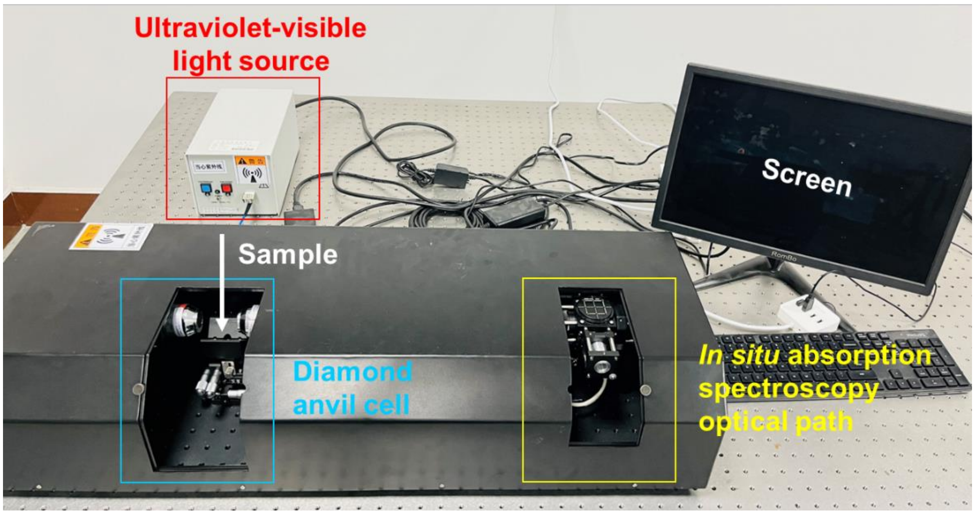

Figure S38: Photograph of in situ high pressure UV absorption spectroscopy system.

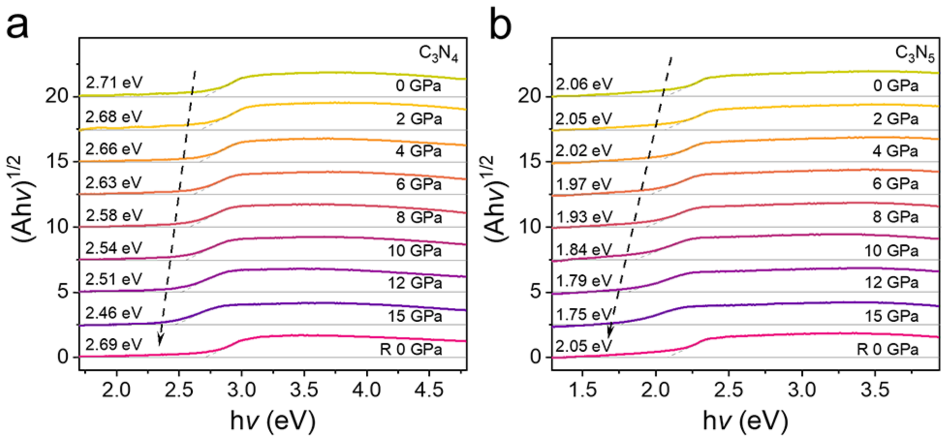

Figure S39: Tauc plots of C_3N_4 (**a**) and C_3N_5 (**b**) from pressure-dependent UV-Vis absorption spectra.

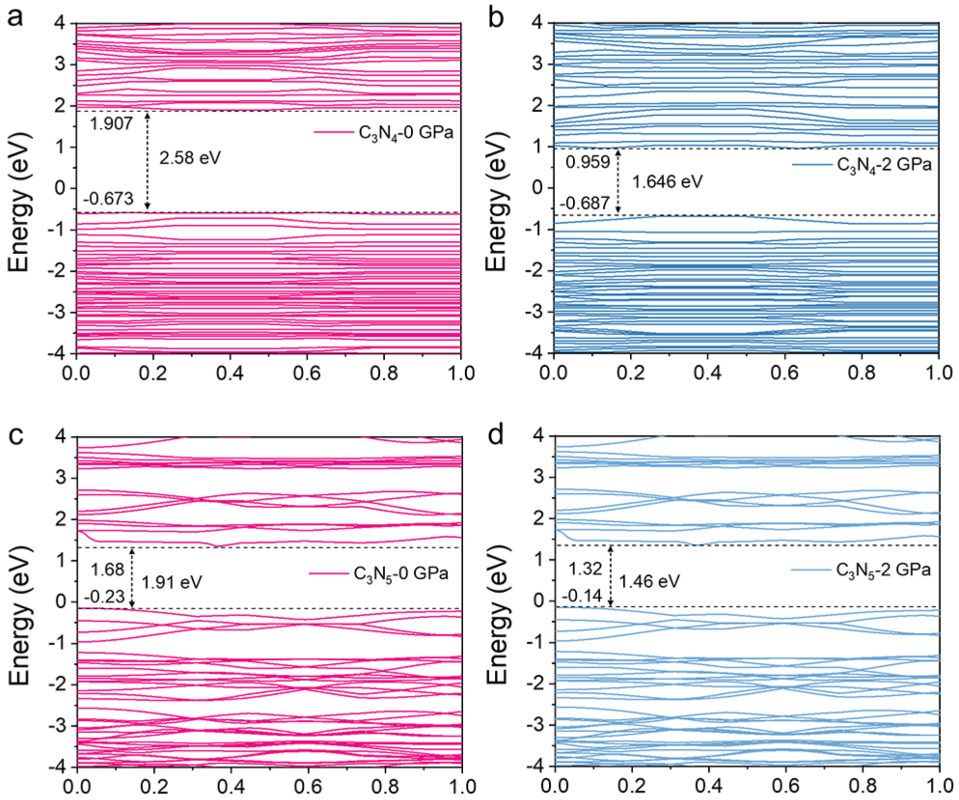

Figure S40: Energy band structures of C_3N_4 (**a** and **b**) and C_3N_5 (**c** and **d**) with 0 and 2 GPa calculated by the HSE06 functional method.

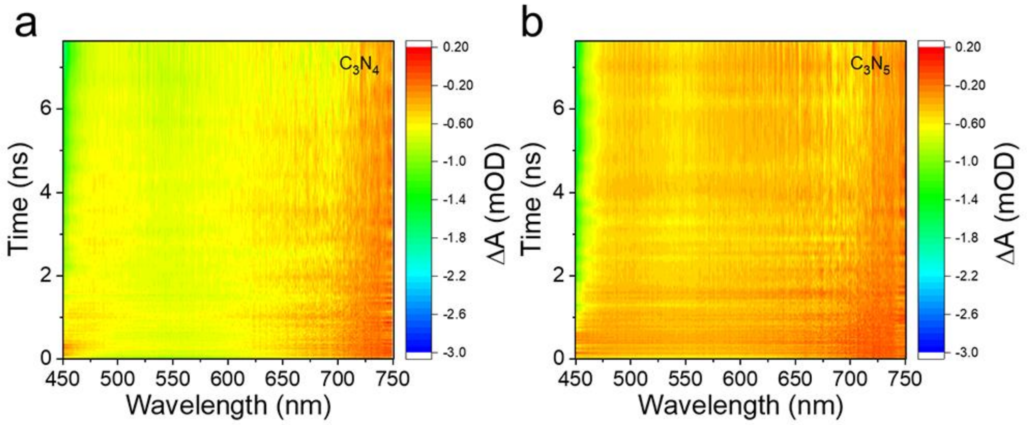

Figure S41: Pseudocolor TA plots of C_3N_4 (**a**) and C_3N_5 (**b**).

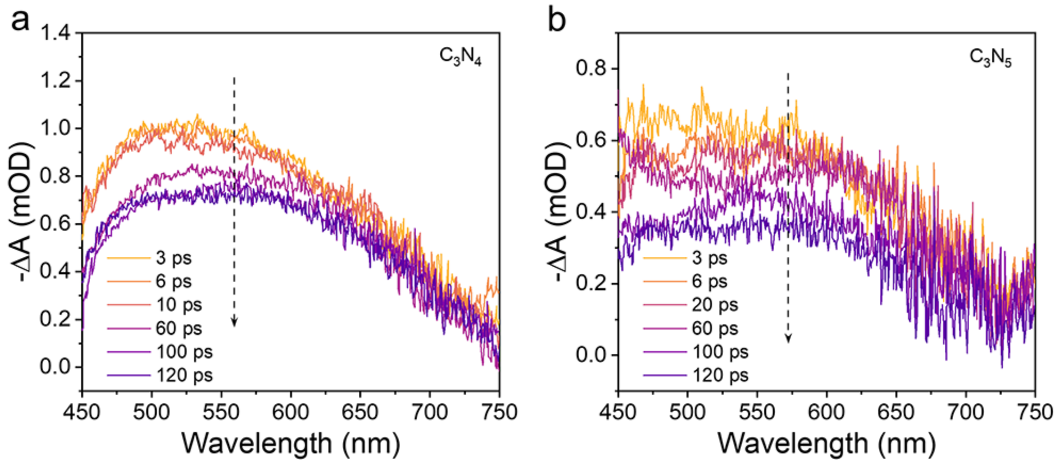

Figure S42: TA spectra at the indicated delay time from 3 ps to 120 ps of C_3N_4 (**a**) and C_3N_5 (**b**).

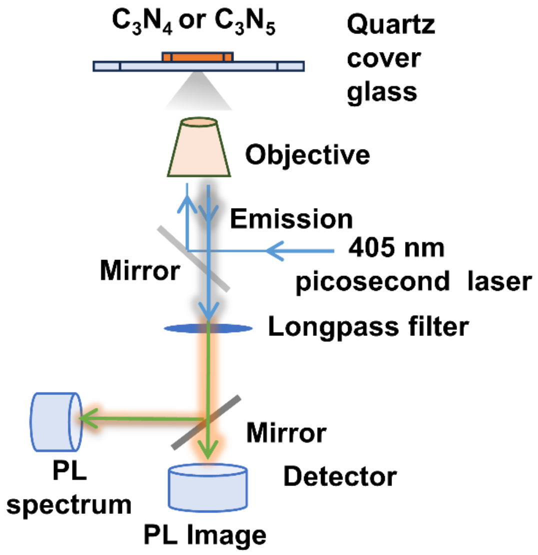

Figure S43: Scheme of the microscopic single-particle PL measurement.

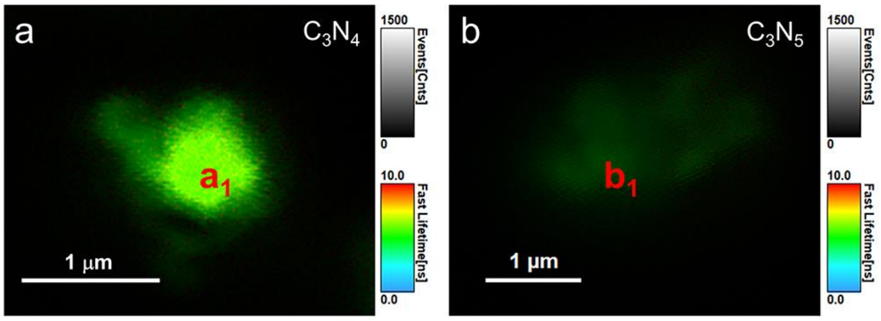

Figure S44: Low-magnification dark-field micrographs of C_3N_4 (**a**) and C_3N_5 (**b**).

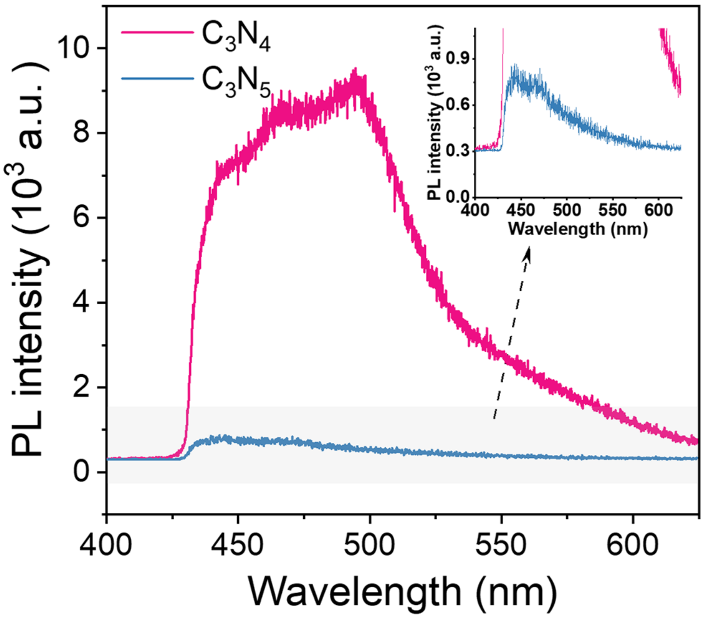

Figure S45: Single-particle PL spectra of C_3N_4 and C_3N_5 by a 405 nm laser. The inset shows enlarged local area.

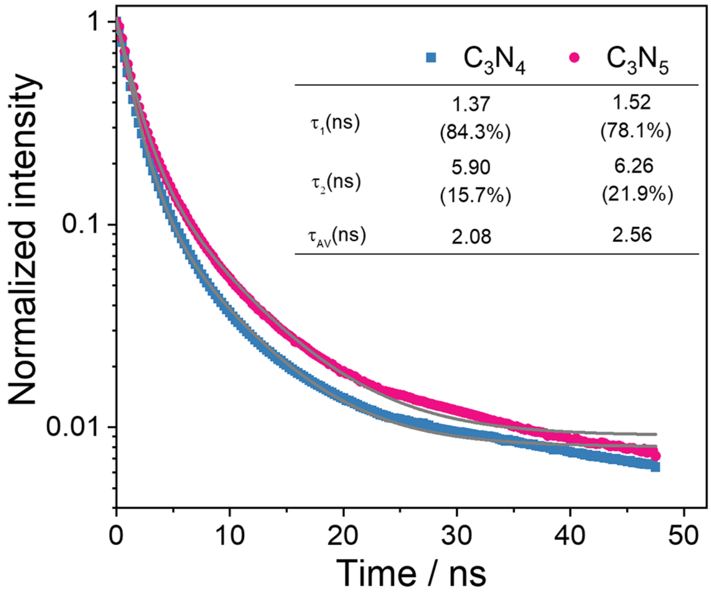

Figure S46: Time-resolved PL spectra of C_3N_4 and C_3N_5. The inset shows obtained values of lifetimes and their fractional components.

Note: Single-particle PL microscope with a picosecond laser at a wavelength of 405 nm was used to probe time-/space-resolved photoelectron generation and transfer of samples with an electron multiplying charge-coupled device (EMCCD) camera (**Figure S43**). The PL emission images observed from confocal microscope system are shown

in **Figure S44**, and several nanosheets with green color were observed in the images, whereas C_3N_4 shows brighter than C_3N_5. Single-particle PL spectra of C_3N_5 (b_1 point in **Figure S44b**) in **Figure S45** displays a significantly weaker peak intensity around range from 450 to 500 nm relative to C_3N_4 (a_1 point in **Figure S44a**), indicating that the introduction of triazole ring on the carbon nitride framework leads to PL quenching with 92.4% efficiency.

The lifetime of excited charged species and charge separation processes of photogenerated carriers using pulsed laser in time-correlated single-photon counting (TCSPC) system for PL decay curves were investigated in **Figure S46**, and the corresponding curves were fitted two-exponentially using **Equations 3** and **4**. The existence of two radiative lifetimes in the fitted PL lifetime spectra of C_3N_4 and C_3N_5 was in good agreement with previously reported carbon nitride based materials.[25] The two components in the PL lifetime decay curve of both g- C_3N_4 and C_3N_5 can be assigned to various energy states in two samples formed by the overlap of C and N sp^2 and sp^3 hybridized orbitals and the presence of lone pairs of electrons, which allow for various radiative transitions.[26] C_3N_4 is composed of tri-s-triazine units interconnected with tertiary nitrogen atoms where C−N sp^3 hybridized state constitute high energy σ and σ* molecular orbitals while C−N sp^2 hybridization gives rise to a conjugated network resulting in low energy π bonding and π* antibonding orbital, which constitutes the valence and conduction bands, respectively.[27] The presence of unbonded lone pairs of electrons on pyridinic N atoms and pyrrolic N in C_3N_5 creates energy levels just below the π bonding orbital and their overlap with the π conjugated system can further decrease the energy of the π molecular orbital resulting in the reduction of the bandgap.[28] The short lifetime components of 1.37 ns with 84.3% contribution in C_3N_4 correspond to charge carrier recombination from σ* and π* antibonding to π MO.[29] The long lifetime component of 5.90 ns with a relative low contribution originated due to intersystem crossing (ISC) of electron from σ* to π* orbital followed by radiative relaxation to conjugated π orbital and trap-assisted radiative recombination.[30] The short lifetime of C_3N_5 at 1.52 ns (78.1%) were slightly

longer lived in comparison to C_3N_4, while the long lifetime at 6.26 ns (21.9%) was significantly increased and the occupation was higher than that of C_3N_4, suggesting that the introduction of triazole ring extends π conjugated network which facilitates better charge carrier mobility on C_3N_5 sheets (delocalized the exciton, as mentioned previously) and prevents faster charge carrier recombination.[24] The average lifetimes of C_3N_4 and C_3N_5 were calculated to be 2.08 and 2.56 ns, respectively. These indicate that the lifetime of photo−generated charge carriers was prolonged after introducing triazole ring induced high dipole moment in C_3N_5, leading to a more effective separation of photogenerated for C_3N_5.

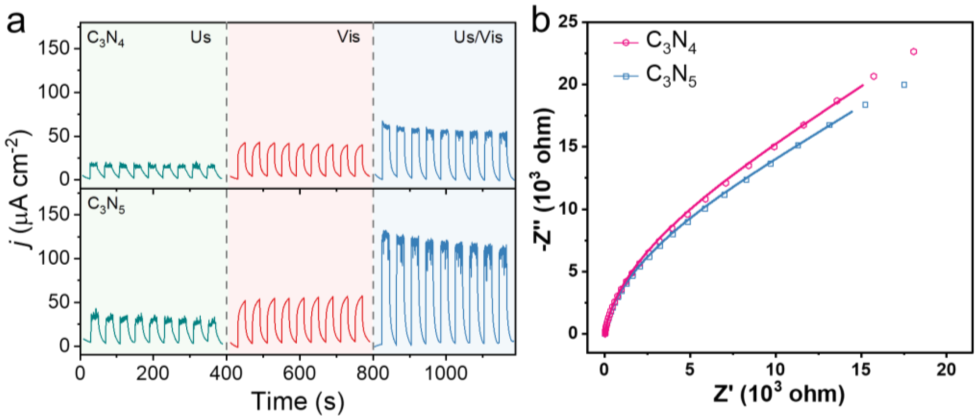

Figure S47: a, Transient current density-time curves of C_3N_4 and C_3N_5 with on-off cycles of US, Vis, and US/Vis at a potential of -0.5 V in 0.5 M Na_2SO_4 solution, **b,** Nyquist plots of C_3N_4 and C_3N_5.

Note: To reveal carriers migration of C_3N_5, the transient piezoelectric current response is depicted in **Figure S47a**, manifesting obvious and repeatable piezo-photo current signals with on/off of applying ultrasound or visible light. As shown in **Figure S47a**, the current intensity (*j*) of C_3N_5 and C_3N_4 in various scenarios follows the sequence C_3N_5/Us/Vis > C_3N_4/Us/Vis > C_3N_5/Vis > C_3N_4/Vis > C_3N_5/Us > C_3N_4/Us. The C_3N_5 under both ultrasound and visible light displays the highest migration rate of carriers, and the result is matched well with H_2O_2 production. The electrochemical impedance spectroscopy (EIS) further explains a high-efficient carriers transfer of C_3N_5 (**Figure S47b**). The arc diameter of C_3N_5 is the smallest than that of C_3N_4. The smaller diameter is due to the lower charge transfer resistance and the faster mobility of electrons,

indicating that the dipole field contributes to highest charges transfer efficiency of C_3N_5. These results fully demonstrate that the dipole field of triazole-based C_3N_5 can rapidly improve the separation rate of carriers.

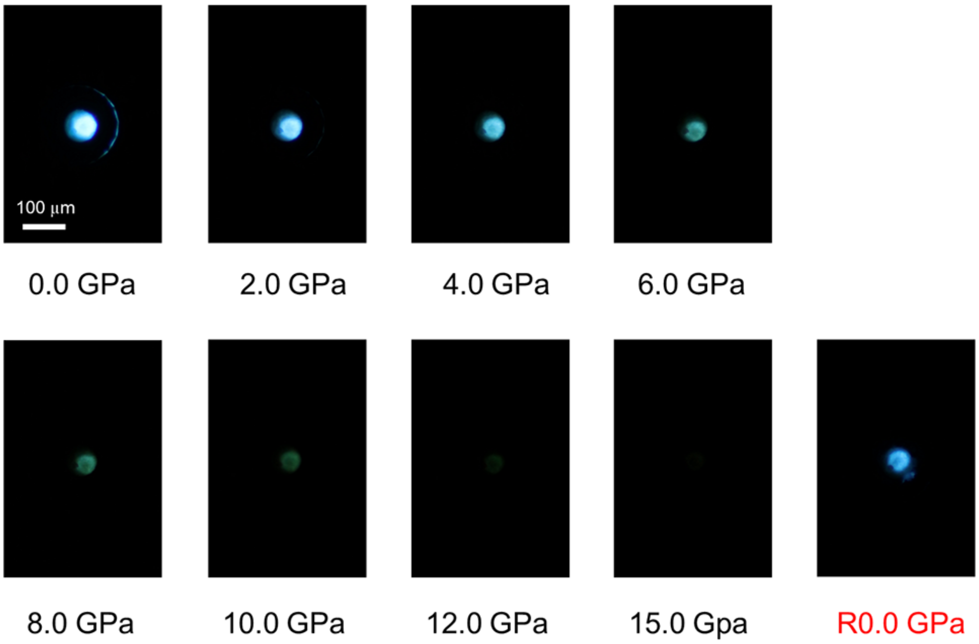

Figure S48: Microphotographs of C_3N_4 at selected pressures irradiated by a 355 nm laser.

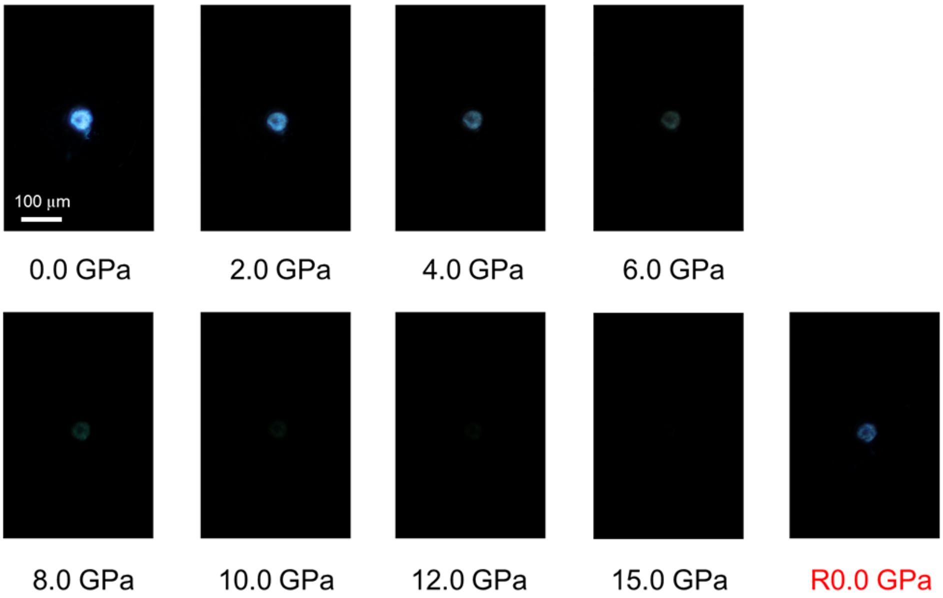

Figure S49: Microphotographs of C_3N_5 at selected pressures irradiated by a 355 nm laser.

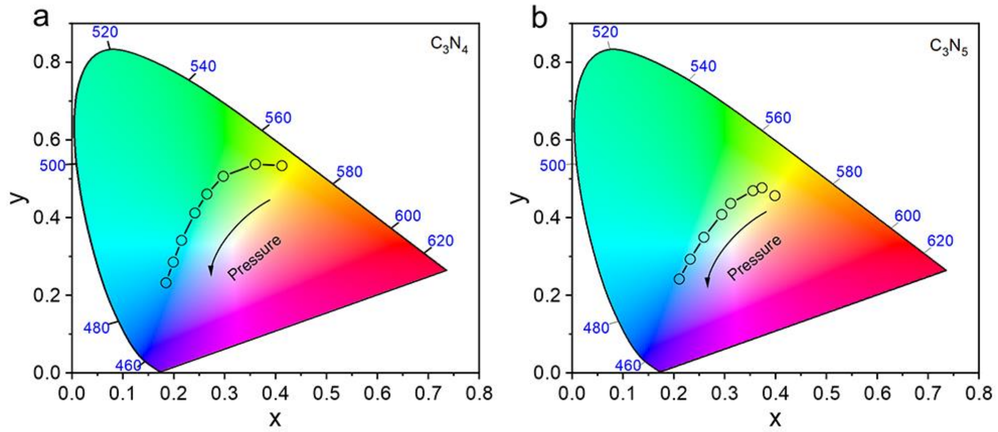

Figure S50: Pressure-dependent chromaticity coordinates of C_3N_4 (**a**) and C_3N_5 (**b**) the emissions from 0 to 15 GPa.

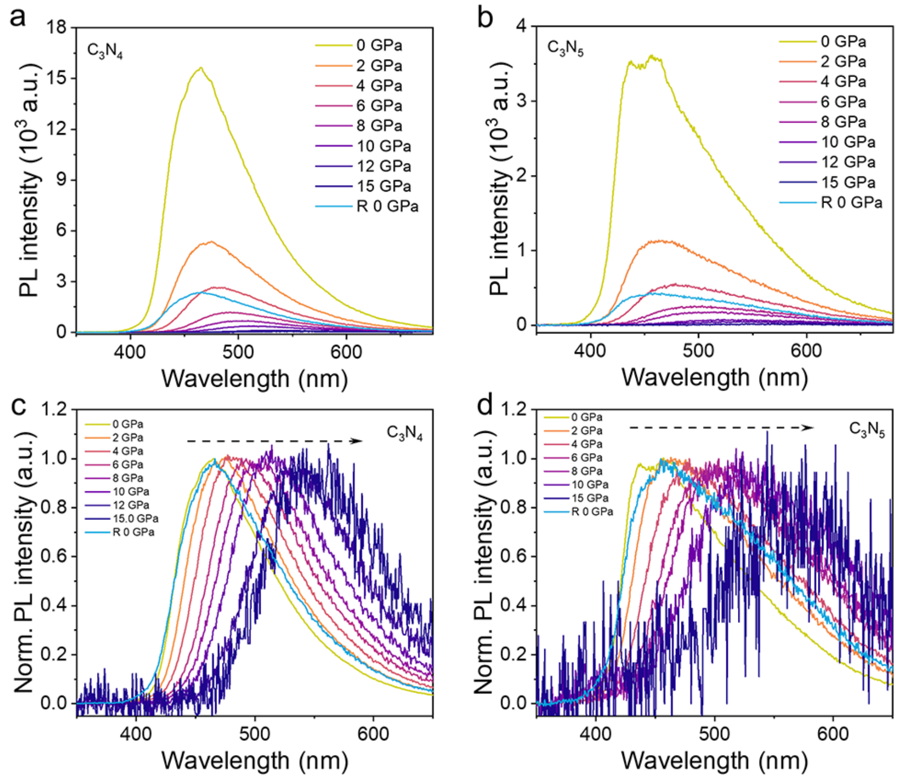

Figure S51: Pressure-dependent (**a** and **b**) and Normalized PL spectra (**c** and **d**) of C_3N_4 and C_3N_5.

Note: As the pressure increases, the layer stacking order of the carbon nitride material decreases while the layer interactions increase. In this case, the tri-s-triazine of carbon nitride shifting to the porous position of the nearest neighboring layer with an obvious

drop in volume and the electron interactions are enhanced, especially, at the positions with larger electronic density where lone pair electrons of nitrogen occur in two samples under high pressure. This should further affect the PL emission related to the lone pair electrons of nitrogen.[31] Hence, we observed that as two samples transforms into a less compressible state, and exhibit a more significant decrease in intensity under pressure.

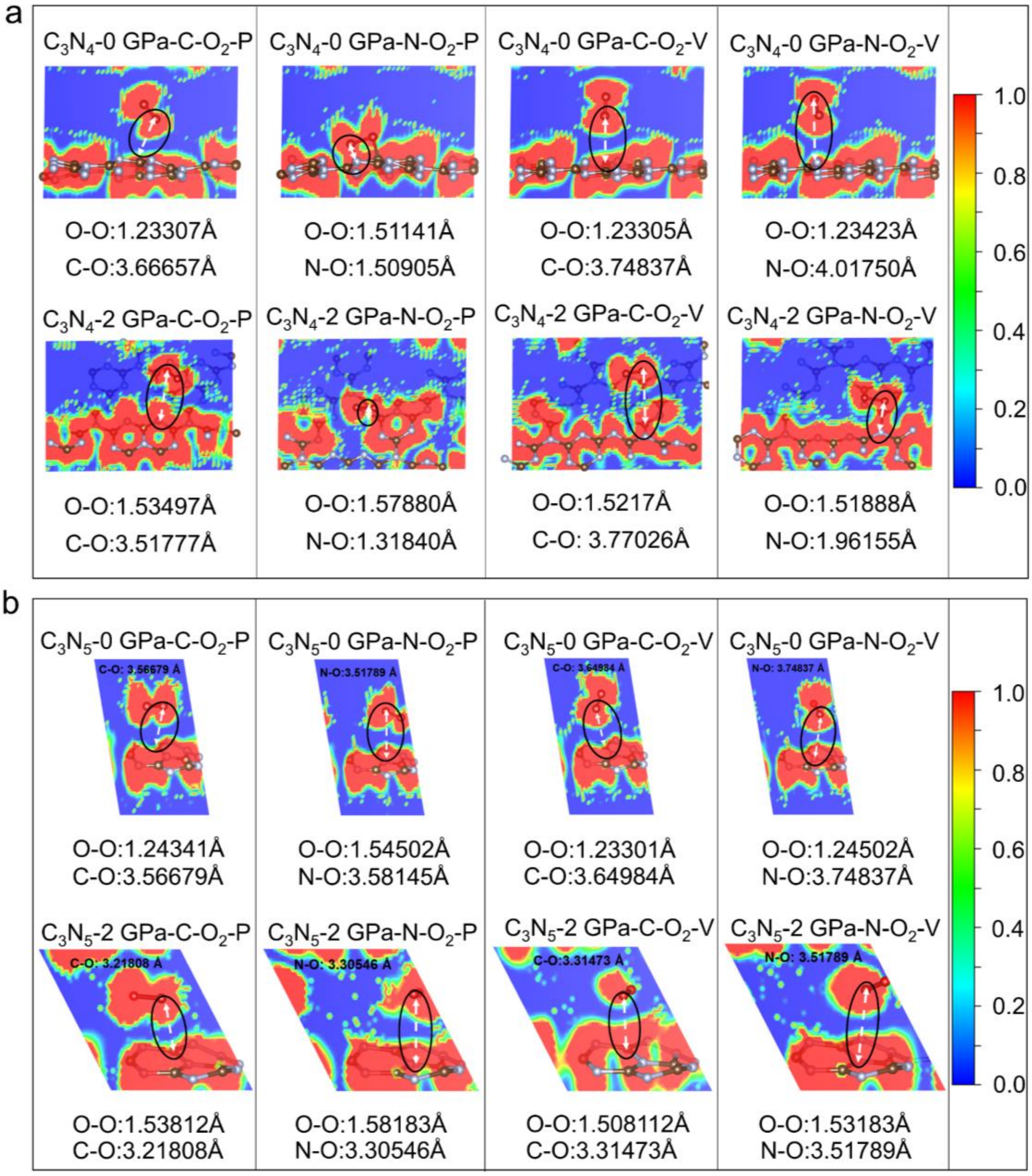

Figure S52: ELF between C_3N_4 (**a**) /C_3N_5 (**b**) and absorbed O_2 (plane and vertical) with 0 and 2 GPa (isosurface level=0.857). The inset also shows bond length of O-O and C-O/N-O.

Note: Electronic local function (ELF) is computed to elucidate the profile of localized distribution of electrons on C_3N_4/C_3N_5 combining with O_2 (plane and vrtical) (**Figures S52a** and **b**). Without pressure, the electron cloud of O_2 is smaller than that with pressure, which reveals an unbound state between O_2 and C_3N_4/C_3N_5 without pressure. By contrast, a stronger electron distribution occurs between O_2 and C_3N_4/C_3N_5 with pressure, proving pressure is able to induce the bonding of O_2 and C_3N_4/C_3N_5 during ORR process. The strong covalent bonds are generated as manifested by high charge density at the interface of C_3N_4/C_3N_5 and O_2 with mechanical pressure, facilitating the combination between adsorbed O_2 and C_3N_4/C_3N_5. For ORR process, the longer bond length of O_2 represents that it is easier to be activated, while the shorter bond length between O_2 and catalyst's atom means O_2 is more likely absorbed on catalyst. Compared to that without pressure, the bond lengths of O-O and C/N-O for all samples almost displays an increasing and a decreasing trend with pressure. These indicate that O_2 is more easily activated and adsorbed on C_3N_4/C_3N_5 under pressure.

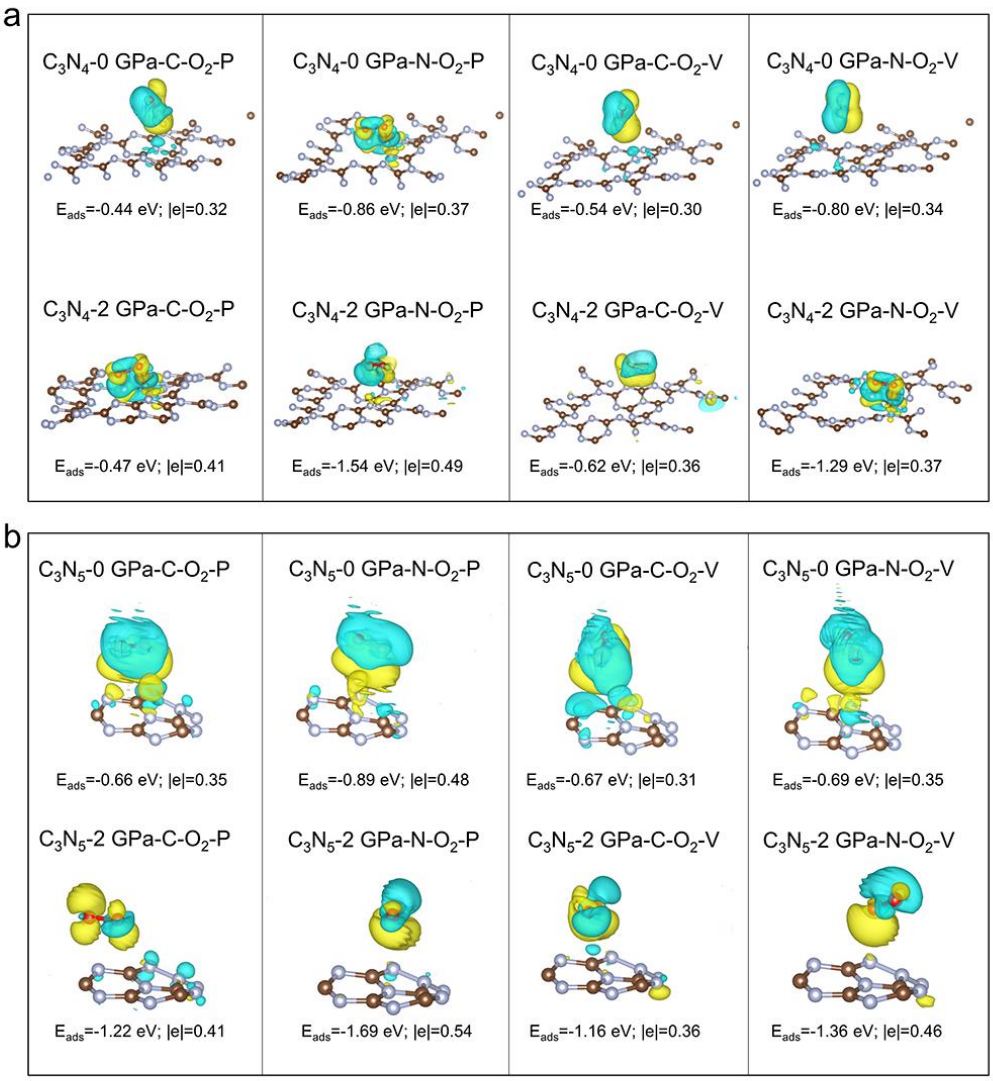

Figure S53: Charge density difference between C_3N_4 (**a**)/C_3N_5 (**b**) and absorbed O_2 (plane and vertical) with 0 and 2 GPa. The yellow and blue colors represent the electron accumulation and depletion at an isosurface value of 0.857 Å e^{-3}.

Note： Figure S53 exhibits the charge density difference and structural changes to describe the detailed reaction between adsorbed O_2 and C_3N_4/C_3N_5. Compared to that without pressure, electron clouds possess a higher density and distribute more intensively between O_2 and C_3N_4/C_3N_5 under pressure. It can be observed that electrons accumulate on O atom and consume on the edge of N more forcefully with applied pressure, verifying the activating effect of N, and C_3N_5-2 GPa-N-P show the excellent electron transfer (|e|=0.54). The phenomena indicate that the pressure contributes to a strong charge interaction between O and N, and to electrons flowing

from C_3N_5 to O_2, helping bond breakage of O_2. To explore interface reactions, the specific active sites of C_3N_4/C_3N_5 for ORR were investigated and analyzed. N atom in C_3N_5 shows the highest adsorption energy (E_{ads}=-1.69 eV) of plane-adsorbed O_2 compared with that of other samples (negative values for E_{ads} indicate heat release). Such stronger O_2 adsorption on N suggests that N atom plays a more crucial role as active sites for converting O_2, which mainly contributes to the following step to generate reducing molecules.

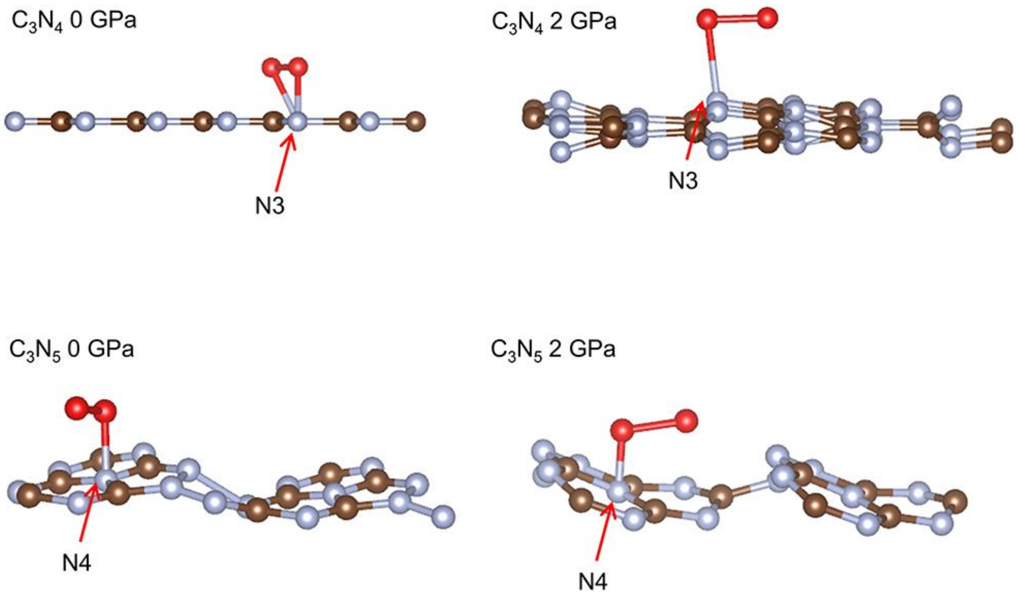

Figure S54: O_2 adsorption on N3 site of C_3N_4 and N4 site of C_3N_5 with 0 and 2 GPa.

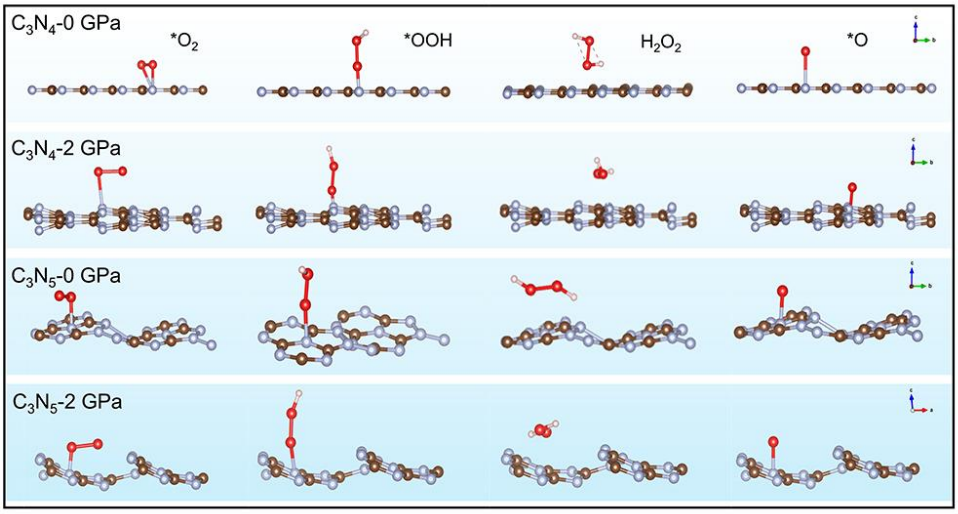

Figure S55: Transition states of ORR for C_3N_4 and C_3N_5 5 with 0 and 2 GPa.

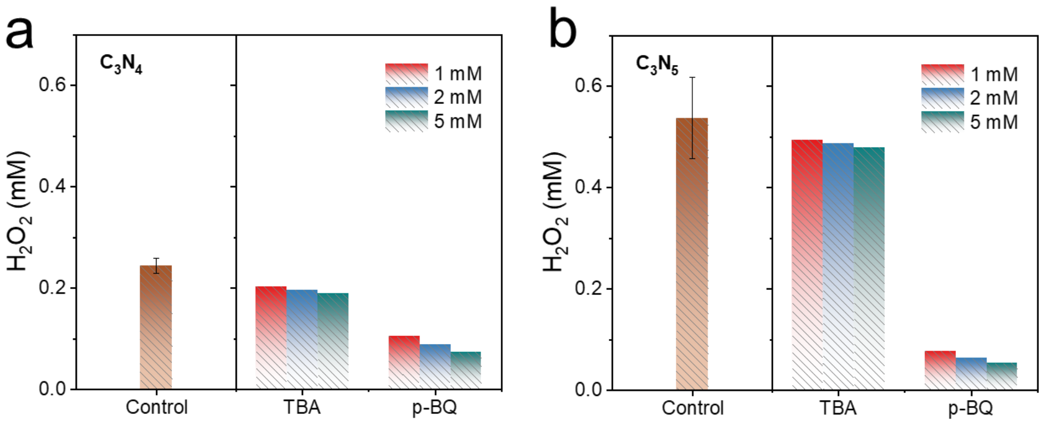

Figure S56: Scavenger tests of C_3N_4 (**a**) and C_3N_5 (**b**) in photocatalytic H_2O_2 production with Us at 60 min (TBA for *OH, p-BQ for $*O_2^-$, C=1, 2, 5 mM).

Note: 1, 2 and 5 mM of scavengers were added to the initial solution in photocatalytic H_2O_2 production with Us. TBA for *OH have little effect on the production of H_2O_2. It is clearly visible, p-BQ for $*O_2^-$) mainly contribute to H_2O_2 production, and with the increase of p-BQ, the production of H_2O_2 gradually decreases.

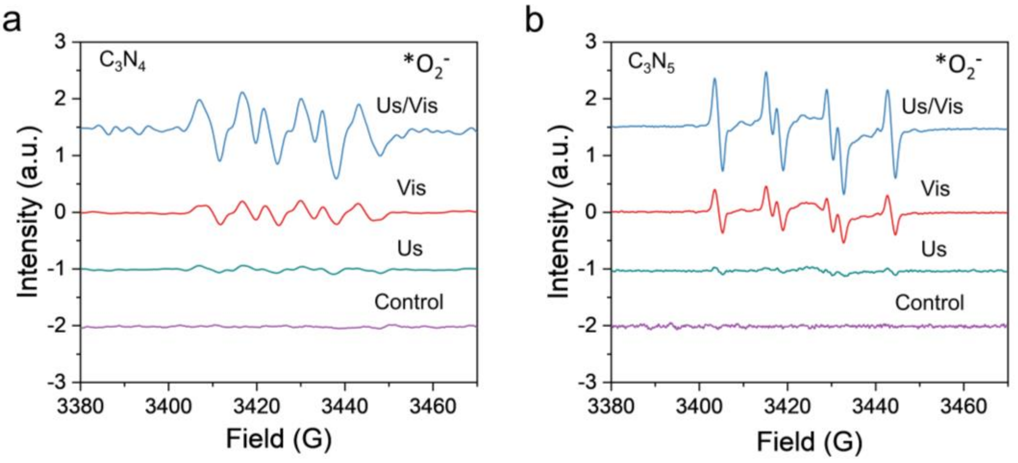

Figure S57: EPR detection of DMPO-$*O_2^-$ in different condition. **a**, C_3N_4. **b**, C_3N_5. Experimental conditions: [catalyst] = 0.5 g L^{-1}, V=100 μL EtOH, $V_{capture\ agent}$=10 μL; US refers to ultrasonic force and Vis refers to visible light.

Note: The signals of C_3N_4 and C_3N_5 present the following sequence: Us/Vis > Vis > Us > Control, and the signal of C_3N_5 is higher than that of C_3N_4.

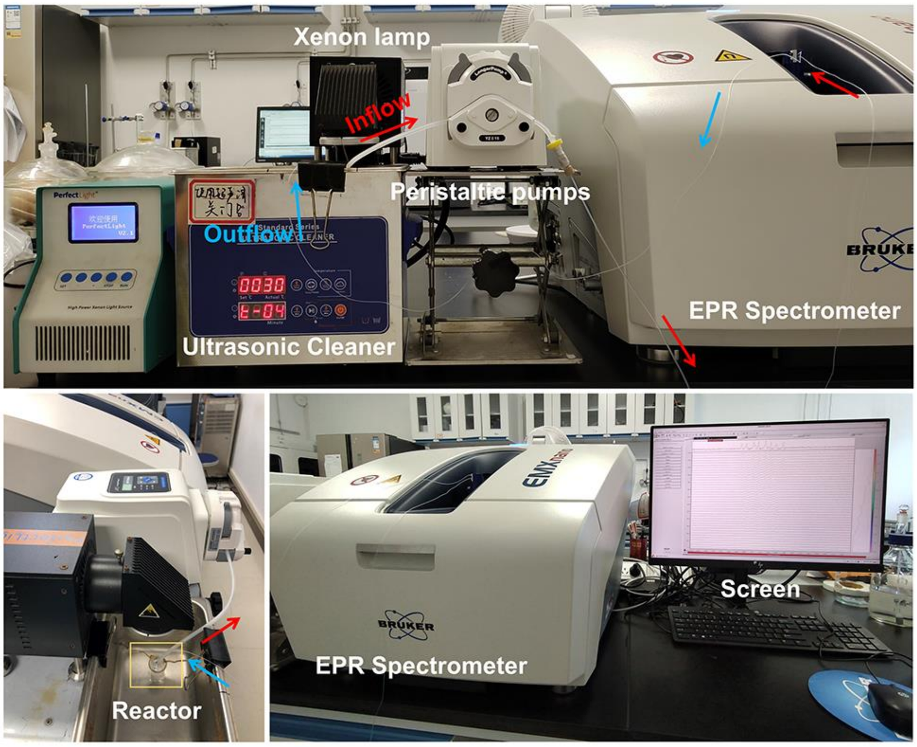

Figure S58: Photograph of *in-situ* EPR system for photocatalytic H_2O_2 production with Us.

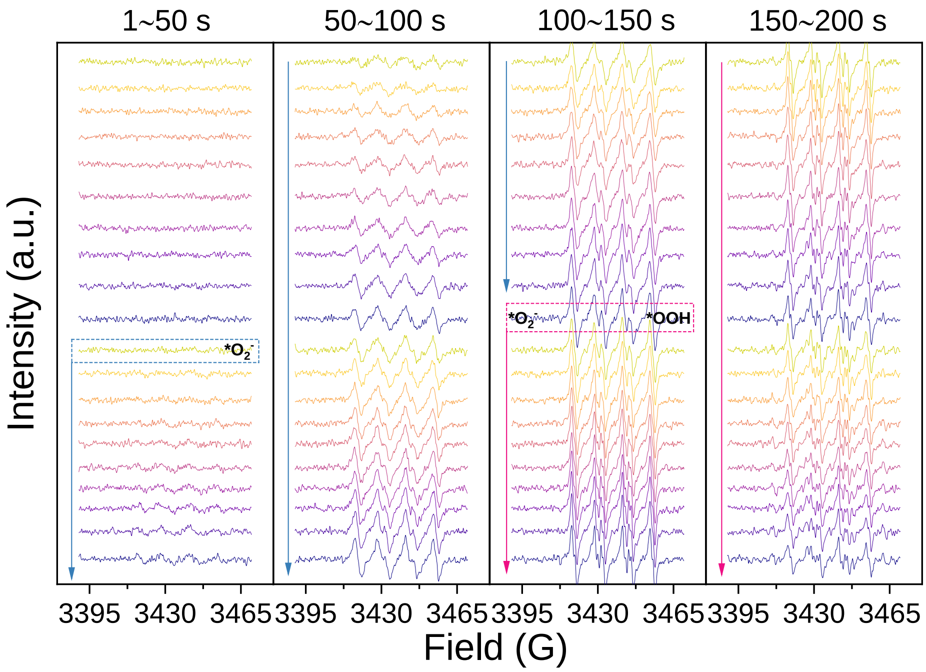

Figure S59: *In-situ* EPR spectrum during H_2O_2 photocatalysis production with Us. Experimental conditions: [catalyst] = 1 g L^{-1}, V=4 mL (10 vol% EtOH), $V_{\text{capture agent}}$=100 µL; US refers to ultrasonic force and Vis refers to visible light.

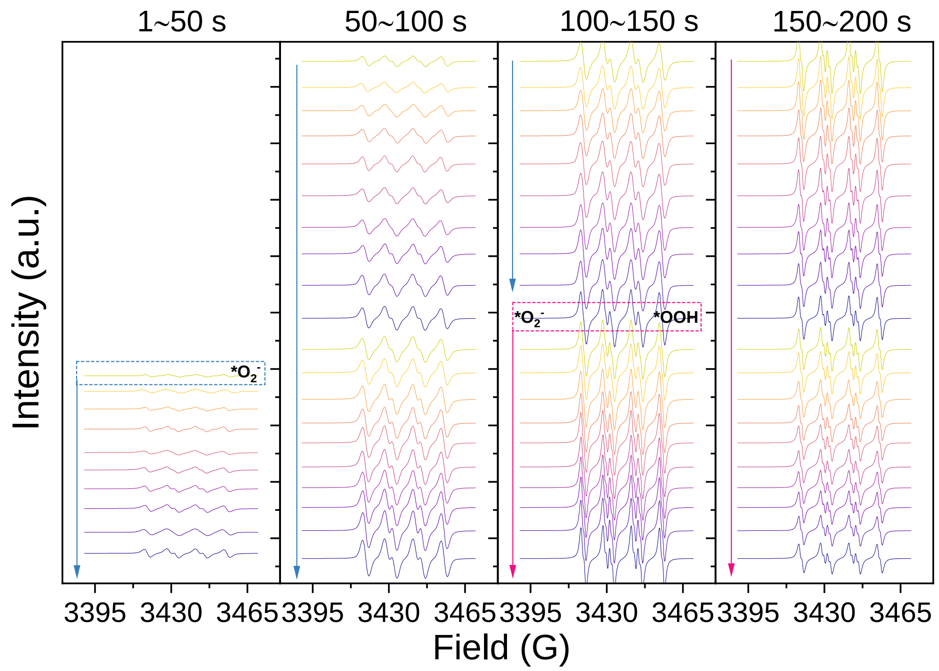

Figure S60: Simulated *in-situ* EPR spectrum during H_2O_2 photocatalysis production with Us.

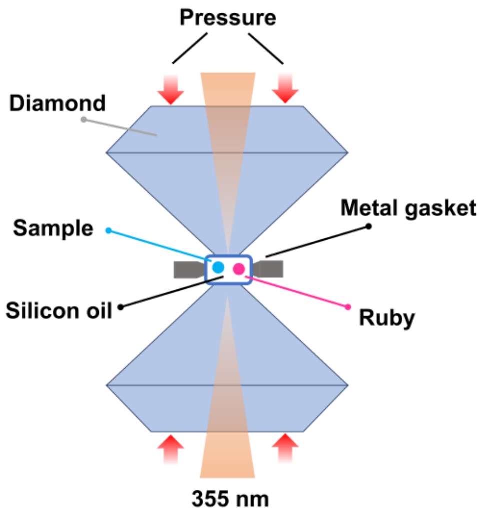

Figure S61: High-pressure experimental equipment DAC device diagram.

Supplementary Tables

Table S1. The relative percentages (at%) of different peaks from C 1s XPS spectra.

Groups	N_2-C=N	C-NH_2	Adv. C	π-π*
C_3N_5	42.0% (287.9 eV)	5.2% (289.1 eV)	46.9%/4.2% (284.8/285.7 eV)	1.7% (293.3 eV)
C_3N_4	32.9% (287.5 eV)	4.8% (289.0 eV)	58.9%/2.7% (284.8/285.9 eV)	0.7% (293.1 eV)

Table S2. The relative percentages (at%) of different peaks from N 1s XPS spectra.

Groups	N-C_3	pyrrolic N	C-N=C	N-H	π-π*
C_3N_5	20.9% (399.2 eV)	12.4% (400.6 eV)	55.5% (398.4 eV)	5.7% (401.2 eV)	5.5% (404.3 eV)
C_3N_4	11.7% (399.7 eV)	- -	66.4% (398.5 eV)	15.5% (400.9 eV)	6.4% (404.3 eV)

Table S3. Elemental composition of 3-AT, CN-200 °C, CN-300 °C, CN-400 °C and C_3N_5 (CN-500°C) as determined by organic elemental analysis (C, N, H).

Sample	Nitrogen (wt%)	Carbon (wt%)	Hydrogen (wt%)	Carbon/ Nitrogen (Atomic ratio)
**3-AT**	66.8	28.8	6.76	0.503
	66.8	28.7	6.29	0.501
	66.7	28.7	5.54	0.502
**CN-200°C**	66.8	28.9	5.05	0.505
	66.5	28.7	5.77	0.504
	66.1	28.8	5.33	0.508
**CN-300 °C**	64.8	29.1	3.95	0.524
	64.8	29.2	4.86	0.526
	64.7	29.3	4.84	0.528
**CN-400 °C**	63.9	31.9	4.4	0.582
	63.6	31.7	4.79	0.581
	63.7	31.8	4.08	0.582
**C_3N_5 (CN-500°C)**	63.1	33.1	3.21	0.611
	63.1	33.2	3.34	0.613
	63.0	33.0	3.42	0.611

Table S4. H_2O_2 production rates for C_3N_5 in this work compared with representative recently reported work.

Catalysts	Condition	Light intensity	Ultrasound conditions	sacrificial agent	H_2O_2 (μmol g^{-1} h^{-1})	Reference
g-C_3N_4/PDI/rGO	Vis	-	-	-	24.1	32
Ag@U-g-C_3N_4	Vis	100 mW cm^{-2}	-	-	70	33
Sb-SAPC15	Vis	400 mW cm^{-2}	-	-	91	34
DCN	Vis	-	-	20 vol% IPA	96.8	35
CTF-BDDBN	Vis	44.5 mW cm^{-2}	-	-	97	36
ZnPPc-NBCN	Vis	100 mW cm^{-2}	-	10 vol% IPA	114	21
Co_1/AQ/C_3N_4	AM-1.5G	100 mW cm^{-2}	-	-	124	37
RF523	Vis	100 mW cm^{-2}	-	-	160	38
PEI/C_3N_4	AM-1.5G	100 mW cm^{-2}	-	-	208.1	39
(K,P,O)-g-C_3N_4	Vis	726.8 mW cm^{-2}	-	10 vol% EtOH	485.71	40
BP/CN	Vis	-	-	10 vol% IPA	540	41
CN_4	Vis	-	-	10 vol% IPA	574	42
KPF_6/g-C_3N_4	Vis	-	-	10 vol% EtOH	600	43
Nv–C≡N–CN	Vis	40 mW cm^{-2}	-	10 vol% IPA	3093	44
TP-PCN	Vis	-	-	10 vol% IPA	6530.8	45
ACNN	Vis	-	-	10 vol% IPA	10200	46
NOCN	Simulating sunlight	-	-	10 vol% IPA	11140	47
PCN-NaCA	Simulating sunlight	27 mW cm^{-2}	-	3.5 wt.% Glycerol	18700	48
CNF/SCNF-MS	Us/Stirring	-	45KW	-	62.8	49
BTO NSs	Us	-	180 W, 35 kHz	10 vol% MeOH	125.59	50
Au/$BiVO_4$	Us	-	120 W, 40 kHz	4-CP	344.4	51

BiOCl	Us	-	-	Tris-buffered solution	420	52
SiO_2/PVDF-HFP	Us	-	300 W, 40 kHz	20 vol% EtOH	492	53
BiOCl	Us/Stirring	-	150 W, 53 kHz	-	560	54
C_3N_{5-X}-O	Us	-	-	-	615	55
C_3N_4	Us/Stirring	-	150 W, 53 kHz/ 300rpm	-	680	56
UBTO-OV2	Us	-	300 W, 40 kHz	10 vol% EtOH	1611.2	57
LFZ	Us/Vis	-	180 W, 40 kHz	-	403	58
Bi_4NbO_8Br	Us/Vis	-	280 W, 40 kHz	10 vol% EtOH	792	59
g-C_3N_4/PDI-g-C_3N_4	Us/Vis	-	200 W, 40 kHz	-	1040	60
Bulk-g-C_3N_4	Us/Vis	-	240 W, 40 kHz	0.1 M glucose	1080	61
ZnS/In_2S_3/BTO	Us/Vis	100 mW cm^{-2}	150 W, 40 kHz	5M EtOH	1131.73	62
$BaTiO_3$:Nb/C	Us/Vis	100 mW cm^{-2}	150 W, 40 kHz	10 vol% EtOH	1360	63
BCVF	Us/Vis	-	100 W, 35 kHz	-	3173.53	64
C_3N_5	Us/Vis	100 mW cm^{-2}	100 W, 40 kHz	10 vol% EtOH	1235.16	This work
C_3N_5	Us/Simulating sunlight	115 mW cm^{-2}	100 W, 40 kHz	10 vol% EtOH	3809.52	This work

Table S5. Vertical and plane adsorption energies (eV) of O_2 for each C or N atoms with 0 and 2 GPa.

Reaction sites	C_3N_4-0 GPa	C_3N_4-2 GPa	C_3N_4-0 GPa	C_3N_4-2 GPa
C1-P	-0.57	-0.75	-0.57	-1.18
C2-P	-0.44	-0.47	-0.66	-1.22
C3-P	-0.56	-0.71	-0.54	-1.17
C1-V	-0.5	-0.51	-0.55	-1.15
C2-V	-0.54	-0.62	-0.67	-1.16
C3-V	-0.52	-0.59	-0.51	-1.13
N1-P	-0.75	-1.33	-0.75	-1.21
N2-P	-0.82	-1.39	-0.81	-1.65
N3-P	-0.86	-1.54	-0.85	-1.07
N4-P	-	-	-0.89	-1.69
N1-P	-0.72	-1.14	-0.52	-1.1
N2-P	-0.77	-1.31	-0.65	-1.36
N3-P	-0.8	-1.35	-0.67	-1.04
N4-P	-	-	-0.69	-1.36

Supplementary References

1. Pi S-Y*, et al.* Fabrication of polypyrrole nanowire arrays-modified electrode for point-of-use water disinfection via low-voltage electroporation. *Water Res.* **207**, 117825 (2021).
2. Han G-F*, et al.* Building and identifying highly active oxygenated groups in carbon materials for oxygen reduction to H_2O_2. *Nat. Commun.* **11**, 2209 (2020).
3. Chen S*, et al.* Chemical identification of catalytically active sites on oxygen-doped carbon nanosheet to decipher the high activity for electro-synthesis hydrogen peroxide. *Angew. Chem. Int. Ed.* **60**, 16607-16614 (2021).
4. Chen S*, et al.* Identification of the highly active Co–N_4 coordination motif for selective oxygen reduction to hydrogen peroxide. *J. Am. Chem. Soc.* **144**, 14505–14516 (2022).
5. Kresse G, Furthmüller J. computer code VASP, Vienna, Austria, 1999. *Comput. Mater. Sci.* **6**, 15 (1996).
6. Han L*, et al.* Environment friendly and remarkably efficient photocatalytic hydrogen evolution based on metal organic framework derived hexagonal/cubic In_2O_3 phase-junction. *Appl. Catal. B: Environ.* **282**, 119602 (2021).
7. He J*, et al.* Breaking the intrinsic activity barriers of perovskite oxides photocatalysts for catalytic CO_2 reduction via piezoelectric polarization. *Appl. Catal. B: Environ.* **317**, 121747 (2022).
8. Fu X, Belwal T, Cravotto G, Luo Z. Sono-physical and sono-chemical effects of ultrasound: Primary applications in extraction and freezing operations and influence on food components. *Ultrason. Sonochem.* **60**, 104726 (2020).
9. Ma J, et al. Piezo-Electrocatalysis for CO_2 Reduction Driven by Vibration. *Adv. Energy Mater.* **12**, 2200253 (2022).
10. Flannigan DJ, Suslick KS. Plasma formation and temperature measurement during single-bubble cavitation. *Nature* **434**, 52-55 (2005).
11. Dontsova D*, et al.* Triazoles: a new class of precursors for the synthesis of negatively charged carbon nitride derivatives. *Chem. Mater.* **27**, 5170-5179 (2015).
12. Niu P, Qiao M, Li Y, Huang L, Zhai T. Distinctive defects engineering in graphitic carbon nitride for greatly extended visible light photocatalytic hydrogen evolution. *Nano Energy* **44**, 73-81 (2018).
13. Wu C*, et al.* Mesoporous Polymeric Cyanamide-Triazole-Heptazine Photocatalysts for Highly-Efficient Water Splitting. *Small* **16**, 2003162 (2020).
14. Han Q, Wang B, Gao J, Qu L. Graphitic carbon Nitride/Nitrogen-rich carbon nanofibers: highly efficient photocatalytic hydrogen evolution without cocatalysts. *Angew. Chem. Int. Ed.* **55**, 10849-10853 (2016).
15. Wang Y*, et al.* Facile synthesis of oxygen doped carbon nitride hollow microsphere for photocatalysis. *Appl. Catal. B: Environ.* **206**, 417-425 (2017).
16. Zhang P*, et al.* Heteroatom dopants promote two-electron O_2 reduction for photocatalytic production of H_2O_2 on polymeric carbon nitride. *Angew. Chem. Int. Ed.* **59**, 16209-16217 (2020).

17. Dippold AA, Klapötke TM. Nitrogen-rich bis-1, 2, 4-triazoles—a comparative study of structural and energetic properties. *Chem.–A Eur. J.* **18**, 16742-16753 (2012);

18. Yin P, Zhang J, He C, Parrish DA, Jean'ne MS. Polynitro-substituted pyrazoles and triazoles as potential energetic materials and oxidizers. *J. Mater. Chem. A* **2**, 3200-3208 (2014).

19. Xiang Q, Yu J, Jaroniec M. Preparation and enhanced visible-light photocatalytic H_2-production activity of graphene/C_3N_4 composites. *J. Phys. Chem. C* **115**, 7355-7363 (2011).

20. Kim IY, et al. Ordered mesoporous C_3N_5 with a combined triazole and triazine framework and its graphene hybrids for the oxygen reduction reaction (ORR). *Angew. Chem. Int. Ed.* **57**, 17135-17140 (2018).

21. Ye Y-X*, et al.* Highly efficient photosynthesis of hydrogen peroxide in ambient conditions. *P. Natl. Acad. Sci.* **118**, e2103964118 (2021).

22. Wu Q*, et al.* A metal-free photocatalyst for highly efficient hydrogen peroxide photoproduction in real seawater. *Nat. Commun.* **12**, 483 (2021).

23. Ke J*, et al.* Facile assembly of Bi_2O_3/Bi_2S_3/MoS_2 np heterojunction with layered n-Bi_2O_3 and p-MoS_2 for enhanced photocatalytic water oxidation and pollutant degradation. *Appl. Catal. B: Environ.* **200**, 47-55 (2017).

24. Luo S*, et al.* $CuInS_2$ quantum dots embedded in Bi_2WO_6 nanoflowers for enhanced visible light photocatalytic removal of contaminants. *Appl. Catal. B: Environ.* **221**, 215-222 (2018).

25. Yang P, Ou H, Fang Y, Wang X. A facile steam reforming strategy to delaminate layered carbon nitride semiconductors for photoredox catalysis. *Angew. Chem. Int. Ed.* **56**, 3992-3996 (2017).

26. Kumar P*, et al.* C_3N_5: a low bandgap semiconductor containing an azo-linked carbon nitride framework for photocatalytic, photovoltaic and adsorbent applications. *J. Am. Chem. Soc.* **141**, 5415-5436 (2019).

27. Sun C*, et al.* Bond contraction and lone pair interaction at nitride surfaces. *J. Appl. Phys.* **90**, 2615-2617 (2001).

28. Niu P, Liu G, Cheng H-M. Nitrogen vacancy-promoted photocatalytic activity of graphitic carbon nitride. *J. Phys. Chem. C* **116**, 11013-11018 (2012).

29. Chen LC, Teng CY, Lin CY, Chang HY, Chen SJ, Teng H. Architecting nitrogen functionalities on graphene oxide photocatalysts for boosting hydrogen production in water decomposition process. *Adv. Energy Mater.* **6**, 1600719 (2016).

30. Liang Q, Li Z, Huang ZH, Kang F, Yang QH. Holey graphitic carbon nitride nanosheets with carbon vacancies for highly improved photocatalytic hydrogen production. *Adv. Funct. Mater.* **25**, 6885-6892 (2015).

31. Hu K*, et al.* Pressure tuned photoluminescence and band gap in two-dimensional layered g-C_3N_4: the effect of interlayer interactions. *Nanoscale* **12**, 12300-12307 (2020).

32. Kofuji Y*, et al.* Carbon nitride–aromatic diimide–graphene nanohybrids: metal-free photocatalysts for solar-to-hydrogen peroxide energy conversion with 0.2% efficiency. *J. Am. Chem. Soc.* **138**, 10019-10025 (2016).

33. Cai J*, et al.* Crafting Mussel-inspired metal nanoparticle-decorated ultrathin graphitic carbon nitride for the degradation of chemical pollutants and production of chemical

resources. *Adv. Mater.* **31**, 1806314 (2019).

34. Teng Z*, et al.* Atomically dispersed antimony on carbon nitride for the artificial photosynthesis of hydrogen peroxide. *Nat. Catal.* **4**, 374-384 (2021).

35. Shi L*, et al.* Photoassisted construction of holey defective g-C_3N_4 photocatalysts for efficient visible-light-driven H_2O_2 production. *Small* **14**, 1703142 (2018).

36. Chen L*, et al.* Acetylene and diacetylene functionalized covalent triazine frameworks as metal-free photocatalysts for hydrogen peroxide production: a new two-electron water oxidation pathway. *Adv. Mater.* **32**, 1904433 (2020).

37. Chu C*, et al.* Spatially separating redox centers on 2D carbon nitride with cobalt single atom for photocatalytic H_2O_2 production. *P. Natl. Acad. Sci.* **117**, 6376-6382 (2020).

38. Shiraishi Y*, et al.* Resorcinol–formaldehyde resins as metal-free semiconductor photocatalysts for solar-to-hydrogen peroxide energy conversion. *Nat. Mater.* **18**, 985-993 (2019).

39. Zeng X*, et al.* Simultaneously tuning charge separation and oxygen reduction pathway on graphitic carbon nitride by polyethylenimine for boosted photocatalytic hydrogen peroxide production. *Acs Catal.* **10**, 3697-3706 (2020).

40. Moon G-h, Fujitsuka M, Kim S, Majima T, Wang X, Choi W. Eco-friendly photochemical production of H_2O_2 through O_2 reduction over carbon nitride frameworks incorporated with multiple heteroelements. *ACS Catal.* **7**, 2886-2895 (2017).

41. Zheng Y, Yu Z, Ou H, Asiri AM, Chen Y, Wang X. Black phosphorus and polymeric carbon nitride heterostructure for photoinduced molecular oxygen activation. *Adv. Funct. Mater.* **28**, 1705407 (2018).

42. Feng C*, et al.* Synthesis of leaf-vein-like g-C_3N_4 with tunable band structures and charge transfer properties for selective photocatalytic H_2O_2 evolution. *Adv. Funct. Mater.* **30**, 2001922 (2020).

43. Kim S*, et al.* Selective charge transfer to dioxygen on KPF_6-modified carbon nitride for photocatalytic synthesis of H_2O_2 under visible light. *J. Catal.* **357**, 51-58 (2018).

44. Zhang X*, et al.* Unraveling the dual defect sites in graphite carbon nitride for ultra-high photocatalytic H_2O_2 evolution. *Energy Environ. Sci.* **15**, 830-842 (2022).

45. Che H, Gao X, Chen J, Hou J, Ao Y, Wang P. Iodide-induced fragmentation of polymerized hydrophilic carbon nitride for high-performance quasi-homogeneous photocatalytic H_2O_2 production. *Angew. Chem. Int. Ed.* **60**, 25546-25550 (2021).

46. Wu S, Yu H, Chen S, Quan X. Enhanced photocatalytic H_2O_2 production over carbon nitride by doping and defect engineering. *ACS Catal.* **10**, 14380-14389 (2020).

47. Zhao H, et al. Rational design of carbon nitride for remarkable photocatalytic H_2O_2 production. *Chem Catal.* **2**, 1720-1733 (2022).

48. Zhao Y, et al. Mechanistic analysis of multiple processes controlling solar-driven H_2O_2 synthesis using engineered polymeric carbon nitride. *Nat. Commun.* **12**, 3701 (2021).

49. Xu T, Xia Z, Li H, Niu P, Wang S, Li L. Constructing crystalline g-C_3N_4/g-$C_3N_{4-x}S_x$ isotype heterostructure for efficient photocatalytic and piezocatalytic performances. *Energy Environ. Mater.* **6**, e12306 (2023).

50. Tang Q, *et al.* Enhanced piezocatalytic performance of $BaTiO_3$ nanosheets with highly exposed {001} Facets. *Adv. Funct. Mater.* 2202180 (2022).
51. Wei Y, Zhang Y, Geng W, Su H, Long M. Efficient bifunctional piezocatalysis of Au/$BiVO_4$ for simultaneous removal of 4-chlorophenol and Cr (VI) in water. *Appl. Catal. B: Environ.* **259**, 118084 (2019).
52. Yoon J, *et al.* Piezobiocatalysis: ultrasound-driven enzymatic oxyfunctionalization of C–H bonds. *ACS Catal.* **10**, 5236-5242 (2020).
53. Wang L, *et al.* Synergistically active piezoelectrical H_2O_2 production composite film achieved from a catalytically inert PVDF−HFP matrix and SiO_2 fillers. *Chem.–Asian J.* **17**, e202200278 (2022).
54. Shao D, Zhang L, Sun S, Wang W. Oxygen reduction reaction for generating H_2O_2 through a piezo-catalytic process over bismuth oxychloride. *ChemSusChem* **11**, 527-531 (2018).
55. Fu C, et al. Dual-defect enhanced piezocatalytic performance of C_3N_5 for multifunctional applications. *Appl. Catal. B: Environ.* **323**, 122196 (2023).
56. Wang K, Shao D, Zhang L, Zhou Y, Wang H, Wang W. Efficient piezo-catalytic hydrogen peroxide production from water and oxygen over graphitic carbon nitride. *J. Mater. Chem. A* **7**, 20383-20389 (2019).
57. Wang C, Chen F, Hu C, Ma T, Zhang Y, Huang H. Efficient piezocatalytic H_2O_2 production of atomic-level thickness $Bi_4Ti_3O_{12}$ nanosheets with surface oxygen vacancy. *Chem. Eng. J.* **431**, 133930 (2022).
58. Zhang L, *et al.* Self-Assembled $LaFeO_3$/$ZnFe_2O_4$/La_2O_3 ultracompact hybrids with enhanced piezo-phototronic effect for oxygen activation in ambient conditions. *Adv. Funct. Mater.* **32**, 2205121 (2022).
59. Hu C, Huang H, Chen F, Zhang Y, Yu H, Ma T. Coupling piezocatalysis and photocatalysis in Bi_4NbO_8X (X= Cl, Br) polar single crystals. *Adv. Funct. Mater.* **30**, 1908168 (2020).
60. Tang R, *et al.* Unique g-C_3N_4/PDI-g-C_3N_4 homojunction with synergistic piezo-photocatalytic effect for aquatic contaminant control and H_2O_2 generation under visible light. *Appl. Catal. B: Environ.* **303**, 120929 (2022).
61. Hu C, *et al.* Exceptional cocatalyst-free photo-enhanced piezocatalytic hydrogen evolution of carbon nitride nanosheets from strong in-plane polarization. *Adv. Mater.* **33**, 2101751 (2021).
62. Zhou X, Shen B, Zhai J, Conesa JC. High Performance generation of H_2O_2 under piezophototronic effect with multi-layer In_2S_3 nanosheets modified by spherical ZnS and $BaTiO_3$ nanopiezoelectrics. *Small Methods* **5**, 2100269 (2021).
63. Zhou X, *et al.* Efficient production of solar hydrogen peroxide using piezoelectric polarization and photoinduced charge transfer of nanopiezoelectrics sensitized by carbon quantum dots. *Adv. Sci*. 2105792 (2022).
64. Wong KT, *et al.* Interfacial Schottky junctions modulated by photo-piezoelectric band bending to govern charge carrier migration for selective H_2O_2 generation. *Appl. Catal. B: Environ.*, 121581 (2022).
